# AIM2 regulates autophagy to mitigate oxidative stress in aged mice with acute liver injury

**DOI:** 10.1038/s41420-024-01870-2

**Published:** 2024-03-01

**Authors:** Chao Hu, Mengjing Li, Yongzhen Chen, Wei Cheng, Haining Wang, Yiming Zhou, Fengmeng Teng, Tao Ling, Jinshun Pan, Haozhe Xu, Yanan Zheng, Guozhong Ji, Ting Zhao, Qiang You

**Affiliations:** 1 Department of Geriatrics, Medical Center for Digestive Diseases, Second Affiliated Hospital of Nanjing Medical University, Nanjing, 210011 China; 2 Department of general practice, Second Affiliated Hospital of Nanjing Medical University, Nanjing, 210011 China; 3 Affilated Hospital of Nanjing University of Chinese Medicine, Nanjing, 210029 China; 4 Department of Medical Oncology, Fudan University Shanghai Cancer Center, Shanghai, 200032 China

**Keywords:** Autophagy, Hepatotoxicity

## Abstract

The cytoplasmic pattern recognition receptor, absent in melanoma 2 (AIM2), detects cytosolic DNA, activating the inflammasome and resulting in pro-inflammatory cytokine production and pyroptotic cell death. Recent research has illuminated AIM2’s contributions to PANoptosis and host defense. However, the role of AIM2 in acetaminophen (APAP)-induced hepatoxicity remains enigmatic. In this study, we unveil AIM2’s novel function as a negative regulator in the pathogenesis of APAP-induced liver damage in aged mice, independently of inflammasome activation. AIM2-deficient aged mice exhibited heightened lipid accumulation and hepatic triglycerides in comparison to their wild-type counterparts. Strikingly, AIM2 knockout mice subjected to APAP overdose demonstrated intensified liver injury, compromised mitochondrial stability, exacerbated glutathione depletion, diminished autophagy, and elevated levels of phosphorylated c-Jun N-terminal kinase (JNK) and extracellular signal-regulated kinase (ERK). Furthermore, our investigation revealed AIM2’s mitochondrial localization; its overexpression in mouse hepatocytes amplified autophagy while dampening JNK phosphorylation. Notably, induction of autophagy through rapamycin administration mitigated serum alanine aminotransferase levels and reduced the necrotic liver area in AIM2-deficient aged mice following APAP overdose. Mechanistically, AIM2 deficiency exacerbated APAP-induced acute liver damage and inflammation in aged mice by intensifying oxidative stress and augmenting the phosphorylation of JNK and ERK. Given its regulatory role in autophagy and lipid peroxidation, AIM2 emerges as a promising therapeutic target for age-related acute liver damage treatment.

## Introduction

Absent in melanoma 2 (AIM2) is a cytoplasmic pattern recognition receptor that detects cytosolic DNA and activates the inflammasome. It belongs to the AIM2-like receptor (ALR) family, which consists of the C-terminal HIN-200 and N-terminal pyrin domain (PYD). The HIN-200 domain directly binds to dsDNA [1], and the PYD domain interacts with the PYD domain of the inflammatory body junction protein ASC (apoptosis-associated speck-like protein containing a caspase recruitment domain (CARD)). The CARD of ASC connects with the CARD of caspase-1 precursor to create the AIM2 inflammatory body, which further activates caspase-1, causing the production of downstream IL-1β and IL-18 as well as the cleavage of gasdermin D [2]. Under steady-state conditions, AIM2 is in a self-inhibitory state due to the interaction between the PYD and HIN domains [3]. The interaction of dsDNA with HIN domains changes this state, attracting ASC to engage in interactions with free PYD domains and start the oligomerization and inflammatory body stimulation [4]. Although AIM2 inflammatory bodies play a protective role in some infectious diseases, they are usually harmful in some aseptic inflammatory disorders, such as neuroinflammation, atherosclerosis, dermatosis, psoriasis, and liver diseases [5–7]. In addition to its function dependent on these inflammatory bodies, AIM2 inhibits the formation of colorectal tumors, which is independent of its inflammatory body functions [8]. Moreover, a recent study has shown that AIM2 forms a complex with pyrin and ZBP1 to drive PANoptosis (pyroptosis, apoptosis, and necroptosis) and host defense [9].

Drug-induced liver damage (DILI) is a common adverse event in medical practice, and APAP-induced liver damage is a classic example of DILI. Intentional or unintentional overdose of APAP often results in hepatic damage and subsequent liver failure [10]. APAP toxicity occurs through the cytochrome P450-mediated formation of the harmful NAPQI metabolite [11], which depletes hepatic reduced glutathione (GSH) and binds to cellular proteins due to its excess presence [12]. The creation of NAPQI-protein adducts in mitochondria leads to an increase in mitochondrial oxidative stress and dysfunction, eventually resulting in cell necrosis [13]. Furthermore, excessive APAP causes the stimulation and translocation of mitogen-activated protein (MAP) kinase JNK to mitochondria, thus triggering membrane permeability transition (MPT) of the mitochondria. As a result, nuclear DNA damage caused by mitochondrial malfunction results in cell necrosis.

Both NOD-like receptor family pyrin domain-containing 3 (NLRP3) and AIM2 belong to the inflammasome family, playing a pivotal role in immune regulation and the inflammatory response. Multiple distinct studies have underscored the crucial involvement of the NLRP3 inflammasome in APAP-induced acute liver injury and subsequent repair processes [14–16]. Nevertheless, the precise impact of AIM2 on APAP-induced acute liver injury remains to be definitively elucidated. With age, liver cells change under the influence of various stressors and the risk of developing liver diseases [17]. It has been shown that aging aggravates APAP-induced acute liver injury and inflammation [18]. However, the role of AIM2 in APAP hepatoxicity with age has not been defined, and warrants full investigations.

In this study, we compared APAP hepatoxicity in WT and *AIM2*^−/−^ mice of different ages. We investigated the underlying mechanisms by analyzing APAP metabolism, hepatic glutathione levels, mitochondria function, signaling pathways, and hepatic inflammation. Moreover, we treated the mice with rapamycin, an autophagy inducer, to explore the role of AIM2 in APAP-induced liver damage with age. Our study revealed a crucial role of AIM2 in regulating autophagy and lipid peroxidation, emphasizing its significance in mitigating APAP hepatotoxicity with age.

## Results

### AIM2 gene knockout exacerbates acute liver damage induced by APAP in aged mice (>30 Weeks)

**Fig. 1:**
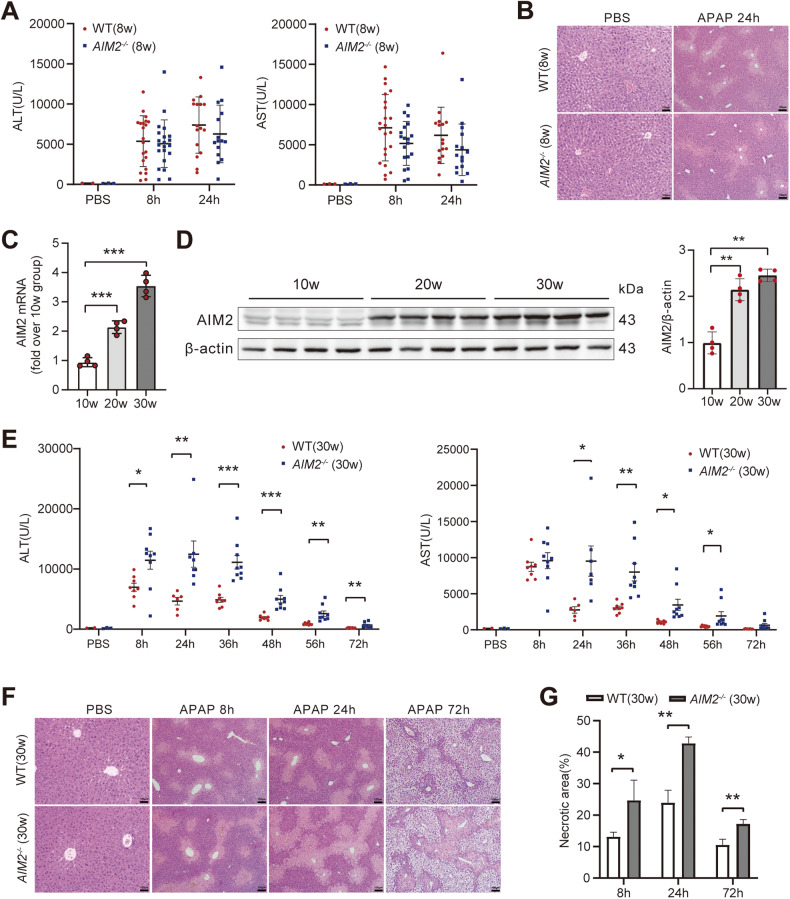
The knockdown of AIM2 exacerbates APAP-induced acute hepatic damage in mice aged 30–32 weeks. **A** Analysis of serum ALT and AST levels and **B** liver H&E staining in 8- and 24-h post i.p. injection of PBS or APAP in 8-week-old WT (*n* = 21) and *AIM2*^−/−^ (*n* = 20) mice. PBS group *n* = 3. The liver H&E staining image is shown with a scale bar of 100 µm. AIM2 levels in the livers of WT mice (10, 20, and 30 weeks old) after APAP administration were quantified using **C** qPCR and **D** Western blotting. *n* = 4 mice per group. **E** Measurement of serum AST and ALT levels at each time point after i.p. injection of PBS or APAP in 30–32-week-old WT (*n* = 8) and *AIM2*^−/−^ (*n* = 9) mice. PBS group *n* = 3. **F**, **G** display liver tissue H&E staining results after 8, 24, and 72 h of APAP administration. The extent of liver injury was quantified using Image J software. Each experimental group comprised 8–10 mice, and the experiment was repeated three times. The statistical analysis showed that AIM2 knockdown aggravated APAP-induced acute hepatic damage in mice (*P < 0.05; **P < 0.01; ***P < 0.001).

To investigate the potential role of AIM2 in acute hepatic injury induced by APAP in mice, we selected eight male WT and eight AIM2-deficient (*AIM2*^−/−^) mice aged 6–8 weeks. Following 15 h of starvation, APAP (300 mg/kg) was intraperitoneally injected to induce acute hepatic damage in the mice. However, no significant differences in liver injury were observed between the WT and *AIM2*^−/−^ mice in this age group (Fig. 1A, B). Research findings have highlighted an age-dependent reduction in AIM2 expression and activation within peripheral blood mononuclear cells (PBMCs) [19]. In our endeavor to delve into whether a similar age-related pattern exists for AIM2 expression in the liver, we conducted an investigation. Through an analysis of both mRNA and protein expression of AIM2 in the livers of mice aged 10, 20, and 30 weeks, we made an unexpected observation: AIM2 expression actually increased with age in the liver, in direct contrast to the diminished expression observed in PBMCs (Fig. 1C, D).

To further examine the potential involvement of AIM2, we conducted the same experiment with male WT and *AIM2*^−/−^ mice aged 30–32 weeks. Our results revealed that compared to WT mice, *AIM2*^−/−^ mice in this age group showed significantly higher serum ALT levels at 8, 24, 36, 48, 56, and 72 h following APAP supplementation (Fig. 1E). Additionally, H&E staining demonstrated a significantly larger area of liver necrosis in the *AIM2*^−/−^ mice compared to WT mice (Fig. 1F, G). Based on these findings, we selected 30–32 weeks old mice for subsequent studies.

### AIM2 deficiency modifies APAP metabolism and enhances inflammatory response in mice

**Fig. 2:**
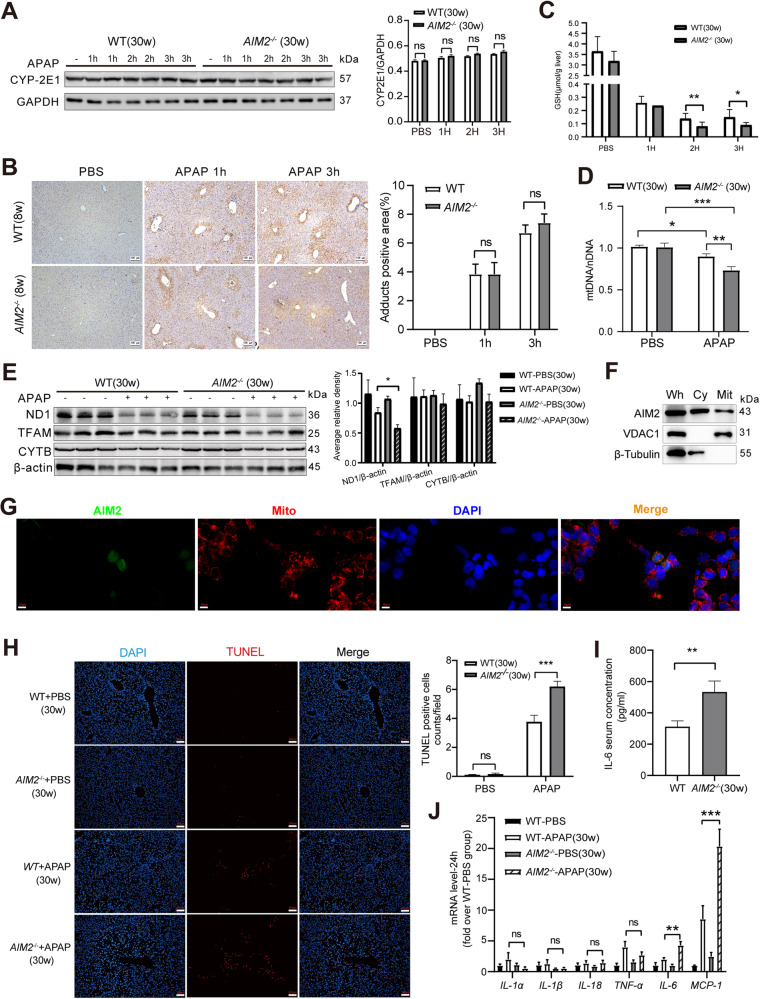
The absence of AIM2 leads to changes in APAP metabolism and enhances inflammation. **A** CYP2E1 protein expression and **B** Immunohistochemical staining of NAPQI-protein adducts were performed, and the images were captured at a 100 µm scale bar. **C** The mtDNA/nDNA content and **D** the expression of ND1, TFAM, and CYTB were measured in the liver tissues after 3 h of PBS or APAP supplementation. **E** Total liver GSH levels were also determined. **F** Cytoplasmic and mitochondrial proteins from 293 T cells were isolated, and AIM2 was analyzed through western blotting. β-tubulin and VDAC1 served as markers for the cytoplasm (Cy) and mitochondria (Mi), respectively. “Wh” designates the whole lysate. **G** Co-localization analysis of AIM2 and mitochondria in 293 T cells. AIM2 is highlighted in green, mitochondria in red, DAPI in blue, and co-localized regions are depicted in yellow. Scale bars, 10 µm. **H** TUNEL staining was conducted 24 h after APAP injection, and the images were captured at a 100 µm scale bar. **I** The serum IL-6 level and **J** the relative hepatic mRNA levels were measured in WT and *AIM2*^−/−^ mice at 24 h after APAP supplementation. Each group consisted of six mice per time point. Statistical analysis indicated no significance (Ns), *P < 0.05, **P < 0.01, and ***P < 0.001.

APAP toxicity is primarily caused by the production of harmful NAPQI metabolites mediated by cytochrome P450 2E1 (CYP2E1) [20, 21], which can result in nitrotyrosine protein adduct formation and mitochondrial DNA damage [22]. Therefore, we investigated the expressions of CYP2E1 and NAPQI-protein adducts in the livers of WT and *AIM2*^−/−^ mice after APAP overdose. Our results showed no significant differences in CYP2E1 protein expression (Fig. 2A) or NAPQI-protein adduct formation between the two strains (Fig. 2B). However, we examined changes in liver GSH levels in WT and *AIM2*^−/−^ mice and found that *AIM2*^−/−^ mice had reduced GSH levels at 2 and 3 h following APAP administration (Fig. 2C). Furthermore, we observed that APAP-treated *AIM2*^−/−^ mice had decreased mitochondrial stability (Fig. 2D) and lower expression of mitochondrial proteins ND1 and TFAM (Fig. 2E) compared to WT mice. Intriguingly, AIM2 was localized in mitochondria (Figs. 2F, G). Moreover, TUNEL assay results revealed a higher number of TUNEL^+^ cells in *AIM2*^−/−^ mice livers at 24 h after APAP injection (Fig. 2H). Additionally, we detected significantly higher levels of the inflammatory factor IL-6 in *AIM2*^−/−^ mice serum at 24 h after APAP administration (Fig. 2I) and a more significant increase in the inflammatory cytokines IL-6 and MCP-1 mRNA levels in *AIM2*^−/−^ mice liver (Fig. 2J). Taken together, these findings suggest that AIM2 deficiency can enhance the inflammatory response in APAP-induced acute liver damage.

### Comparing genetic alterations in WT and *AIM2*^−/−^ mice aged 6–8 and 30–32 weeks following APAP overdose

**Fig. 3:**
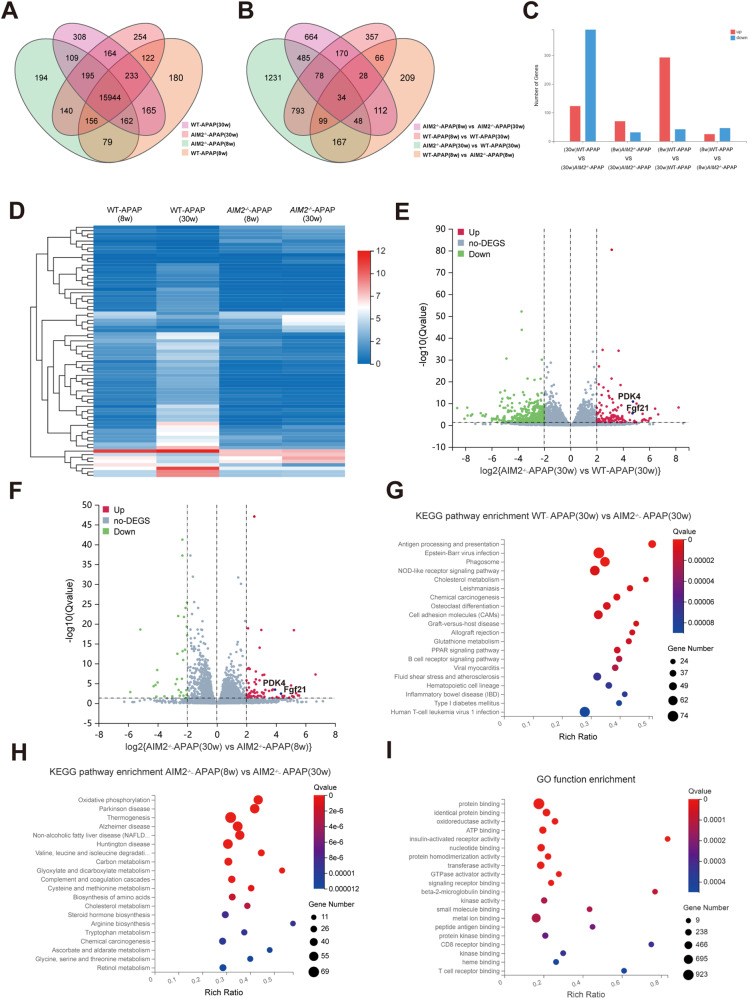
RNA-seq of WT and *AIM2*^−/−^ mice aged 6–8 and 30–32 weeks after APAP treatment. The analysis of RNA-seq data resulted in the Venn diagrams of **A** the total number and **B** the differentially expressed genes (DEGs) in four groups. The statistically significant DEGs were plotted in the four comparisons (**C**) and visualized in the heatmap (**D**), with relative mRNA expression per row (red, high; blue, low). The fold alteration in gene expression (log2FC) and adjusted *P* value (−log10) were plotted in the scatter plot between the livers of WT-APAP (30 w) and *AIM2*^−/−^-APAP (30 w) group (E) and *AIM2*^−/−^-APAP (8w) and *AIM2*^−/−^-APAP (30 w) group (**F**). The KEGG pathways of DEGs in WT-APAP (30 w) and *AIM2*^−/−^-APAP (30 w) group (**G**) and *AIM2*^−/−^-APAP (8w) and *AIM2*^−/−^-APAP (30 w) group (**H**) were ranked by *P* value. The GO functional enrichment of upregulated DEGs in the livers of APAP-treated *AIM2*^−/−^ mice was conducted (**I**), with a false discovery rate (FDR) of <0.05 and ±2 fold change (FC).

To further investigate the reason for the discrepancy in experimental results between 6–8 and 30–32-week-old mice, we conducted RNA-seq on liver tissues from three WT and *AIM2*^−/−^ mice at each age stage after 24 h of APAP stimulation. We detected 18,410 genes, and the average mapping ratio with both the reference genome and gene was 95.26 and 84.12%, respectively (Fig. 3A). There were 513 differentially expressed genes (DEGs) in the WT-APAP (30 w) and *AIM2*^−/−^-APAP (30 w) group (123 upregulated and 390 downregulated), 101 DEGs in the *AIM2*^−/−^-APAP (8w) and *AIM2*^−/−^-APAP (30 w) group (70 upregulated and 31 downregulated). There were 335 DEGs in the WT-APAP (8w) and WT-APAP (30 w) groups (293 upregulated and 42 downregulated), and 71 DEGs in the WT-APAP (8w) and *AIM2*^−/−^-APAP (8w) group (25 upregulated and 46 downregulated) (false discover rate (FDR) <0.05, and ±2 fold change (FC)) (Fig. 3B–D). Previous studies have shown that aging is associated with increased FGF21 levels [23], and PDK4 deficiency protects against APAP-induced hepatotoxicity [24]. Compared to WT-APAP (30 w) and *AIM2*^−/−^-APAP (8w) mice, both PDK4 and Fgf21 were highly expressed in *AIM2*^−/−^-APAP (30 w) mice (Fig. 3E, F). We utilized the Kyoto Encyclopedia of Genes and Genomes (KEGG) to perform an unbiased pathway enrichment analysis of DEGs. The results showed that antigen presentation and processing and cholesterol metabolism signaling pathways were mainly involved in WT-APAP (30 w) and *AIM2*^−/−^-APAP (30 w) groups (Fig. 3G), while oxidative phosphorylation and metabolic pathways were most significant in *AIM2*^−/−^-APAP (8w) and *AIM2*^−/−^-APAP (30 w) groups (Fig. 3H). GO function analysis indicated that liver genes of *AIM2*^−/−^ mice were enriched in response to protein binding and enzyme activity after 24 h of APAP treatment (Fig. 3I).

### Deletion of AIM2 promotes lipid deposition and increases lipid peroxidation

**Fig. 4:**
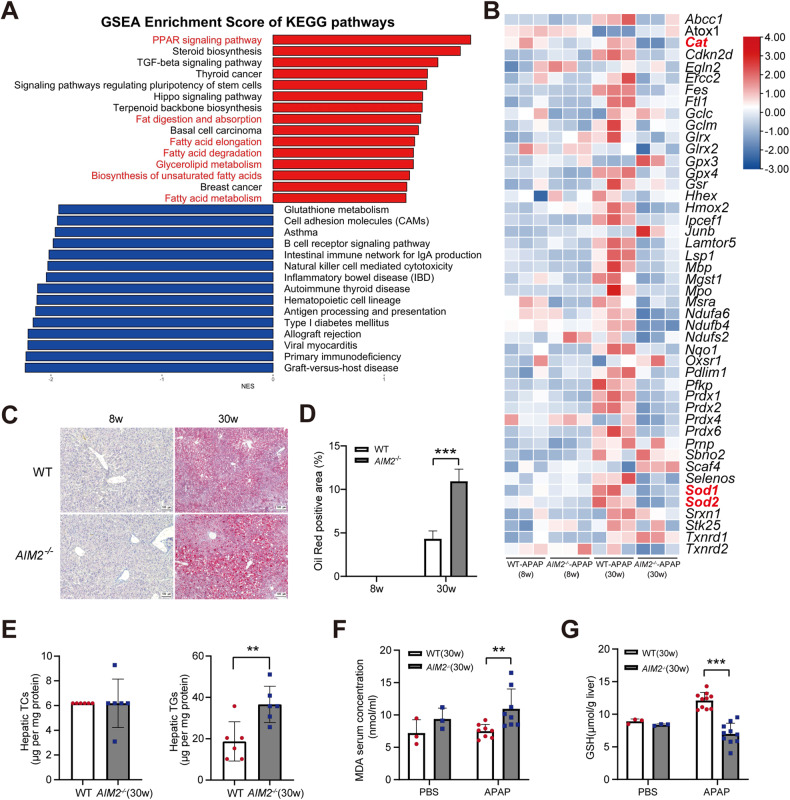
AIM2 deficiency promotes both lipid accumulation and lipid peroxidation. **A** GSEA Enrichment Score of KEGG pathways. **B** The heatmap of DEGs related to the ROS mechanism identified by Hallmark GSEA. Relative mRNA expression per row (red, high; blue, low). **C**, **D** Oil red O staining of liver segments from 6–8 and 30–32-week-old WT and *AIM2*^−/−^ mice. Scale bar, 100 µm. **E** The liver homogenate content of TCs and TGs. *n* = 6 mice per group. **F** The serum MDA content and **G** the total liver GSH level in 30–32-week-old WT and *AIM2*^−/−^ mice supplemented with PBS or APAP for 24 h. *n* = 8–10 mice per group. **P < 0.01; ***P < 0.001.

We observed that most *AIM2*^−/−^ mice exhibited significant weight gain and liver enlargement after 30 weeks (supplementary Fig. 2A, B), with GSEA analysis revealing that 7 of the top 15 enriched KEGG pathways were related to lipid metabolism (Fig. 4A). A heatmap related to lipid metabolism revealed that compared to WT-APAP (30 w) mice, genes associated with Pro-Adipogenesis (such as Fabp4, Irs2, and Jun) and cholesterol synthesis (such as Hmgcs1 and Hmgcs2) were significantly upregulated in the liver of *AIM2*^−/−^-APAP (30 w) mice (supplementary Fig. 2C). Hallmark GSEA analysis of DEGs based on RNA-seq data showed that reactive oxygen species (ROS) pathway activation was reduced in *AIM2*^−/−^-APAP (30 w) compared to WT-APAP (30 w). ROS are eliminated by the action of two important enzymes known as catalase (CAT) and superoxide dismutase (SOD) [25]. A heatmap of the ROS pathway revealed that the expressions of CAT and SOD enzymes were significantly lower in *AIM2*^−/−^-APAP (30 w) than in WT-APAP (30 w) mice (Fig. 4B). This suggests that the ability of *AIM2*^−/−^ mice to handle oxidative stress is weakened after excessive APAP use, leading to liver damage. We hypothesize that lipid accumulation in the liver after AIM2 knockout weakens the anti-oxidative stress ability, leading to increased lipid peroxidation and aggravating hepatic damage. Oil red O staining of liver segments from WT (30 w) and *AIM2*^−/−^ (30 w) mice validated hepatic lipid accumulation, which showed no difference in staining results at eight weeks (Fig. 4C, D). Additionally, we detected the content of cholesterol (TC) and triglyceride (TG) in liver homogenate, which showed significantly elevated hepatic TGs in *AIM2*^−/−^ mice compared to WT mice, but no variation in hepatic TCs (Fig. 4E). Malondialdehyde (MDA) is an important lipid peroxidation product [26]. The content of MDA in serum confirmed our hypothesis that the lipid peroxidation of *AIM2*^−/−^ mice was significantly elevated after one day of APAP supplementation (Fig. 4F). Similarly, compared to WT mice, GSH in the liver of *AIM2*^−/−^ mice was significantly reduced after 24 h of treatment with APAP (Fig. 4G). Overall, these results demonstrate that AIM2 deficiency promotes lipid accumulation and attenuates anti-oxidant stress.

### AIM2 deficiency promotes activation of JNK and ERK signaling pathways

**Fig. 5:**
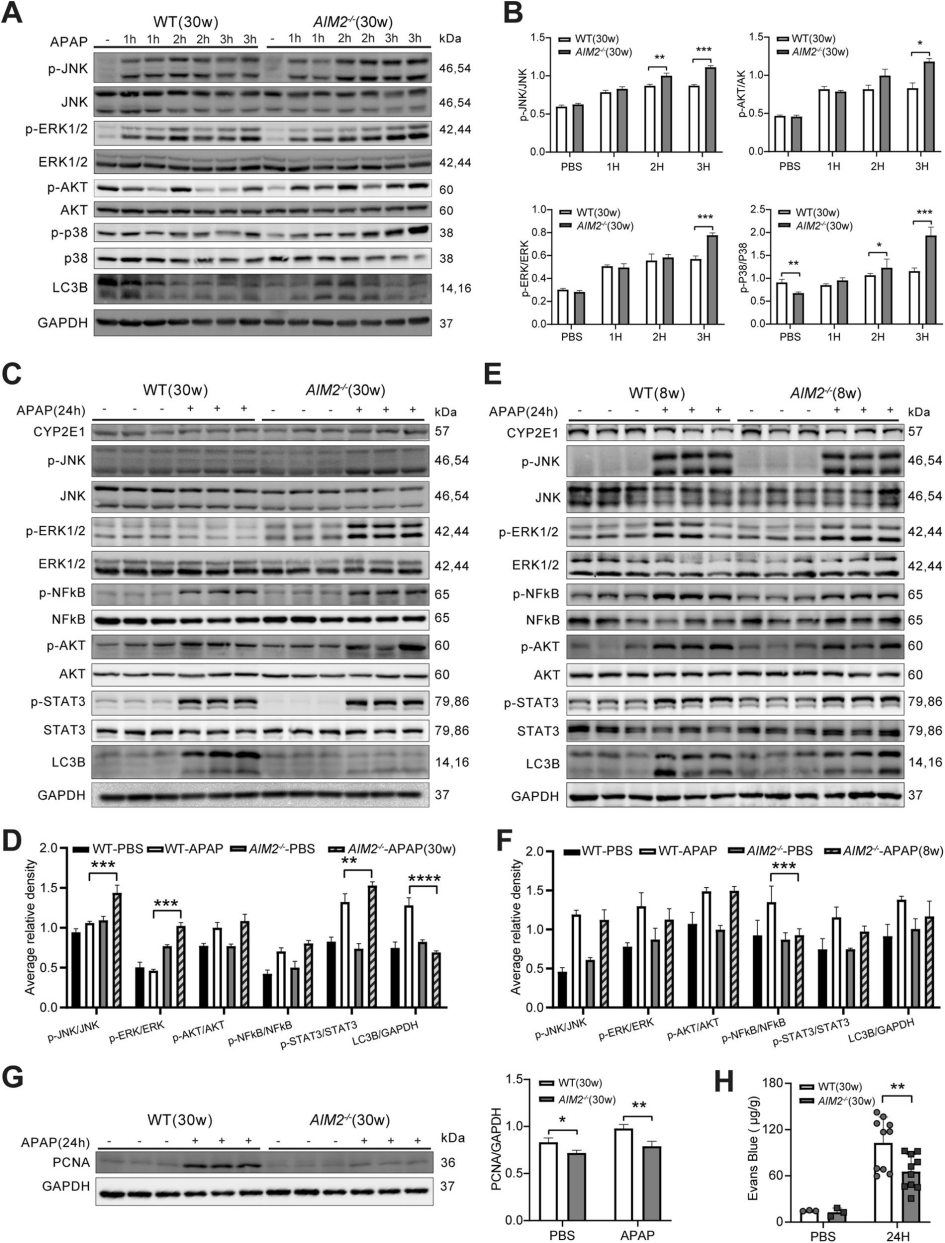
The absence of AIM2 results in increased activation of JNK and ERK pathways. **A**, **B** WT and *AIM2*^−/−^ mice aged 30–32 weeks were administered either PBS or APAP for 1, 2, 3 h, and **C**, **D** for 24 h. **E**, **F** WT and *AIM2*^−/−^ mice aged 6–8 weeks were supplemented with PBS or APAP for 24 h. Protein expression was analyzed using western blotting, and phosphorylated proteins were normalized to total proteins. A representative Western blot from three independent experiments is shown. Immunoblot analysis of PCNA in liver homogenates was conducted (**G**), and quantification of Evans blue dye extravasation was performed at 24 h after APAP injection (**H**). The number of mice in each group ranged from 9 to 10, and the statistical significance levels are indicated as follows: *P < 0.05, **P < 0.01, and ***P < 0.001.

Previous studies have reported that the phosphorylation of c-Jun N-terminal kinase (JNK), a member of the mitogen-activated protein kinase (MAPK) family, can further enhance reactive oxygen species (ROS) formation as a “second hit” after treatment with the drug acetaminophen (APAP) [27]. To investigate the activation of MAPK signaling pathways after APAP treatment, we measured the levels of JNK, ERK, and P38 phosphorylation at 1, 2, 3, and 24 h in both *AIM2*^−/−^ and WT mice. A previous study had shown that 3 h after APAP treatment is the critical time for protein adduct creation [28]. During this period, we found that compared to WT (30 weeks old) mice, *AIM2*^−/−^ (30 weeks old) mice had significantly higher levels of JNK, ERK, and P38 phosphorylation, but no difference in LC3B expression (Fig. 5A, B). After 24 h of APAP administration, the phosphorylated JNK and ERK remained significantly elevated in *AIM2*^−/−^ (30 weeks old) mice compared to WT (30 weeks old) mice. Additionally, the lack of AIM2 led to higher levels of p-SATAT3 and lower levels of LC3B (Fig. 5C, D). We also detected the same indexes of liver protein in 6–8 weeks old mice as controls, and found no difference in p-JNK, p-ERK, and LC3B levels (Fig. 5E, F).

To investigate the impact of AIM2 knockout on liver injury repair, we assessed the expression of proliferating cell nuclear antigen (PCNA) to measure hepatocyte growth. We found that PCNA expression was dramatically downregulated in *AIM2*^−/−^ mice 24 h after APAP administration, suggesting that AIM2 deficiency impairs hepatocyte proliferation following APAP-induced hepatic damage (Fig. 5G). Furthermore, we utilized the Evans Blue assay to determine endothelial vascular permeability [29]. Interestingly, *AIM2*^−/−^ mice exhibited weaker endothelial cell permeability than WT mice following APAP-induced hepatic injury (Fig. 5H).

### AIM2 deficiency promotes ERK signaling activation in macrophages and inhibits autophagy pathway activation in hepatocytes

**Fig. 6:**
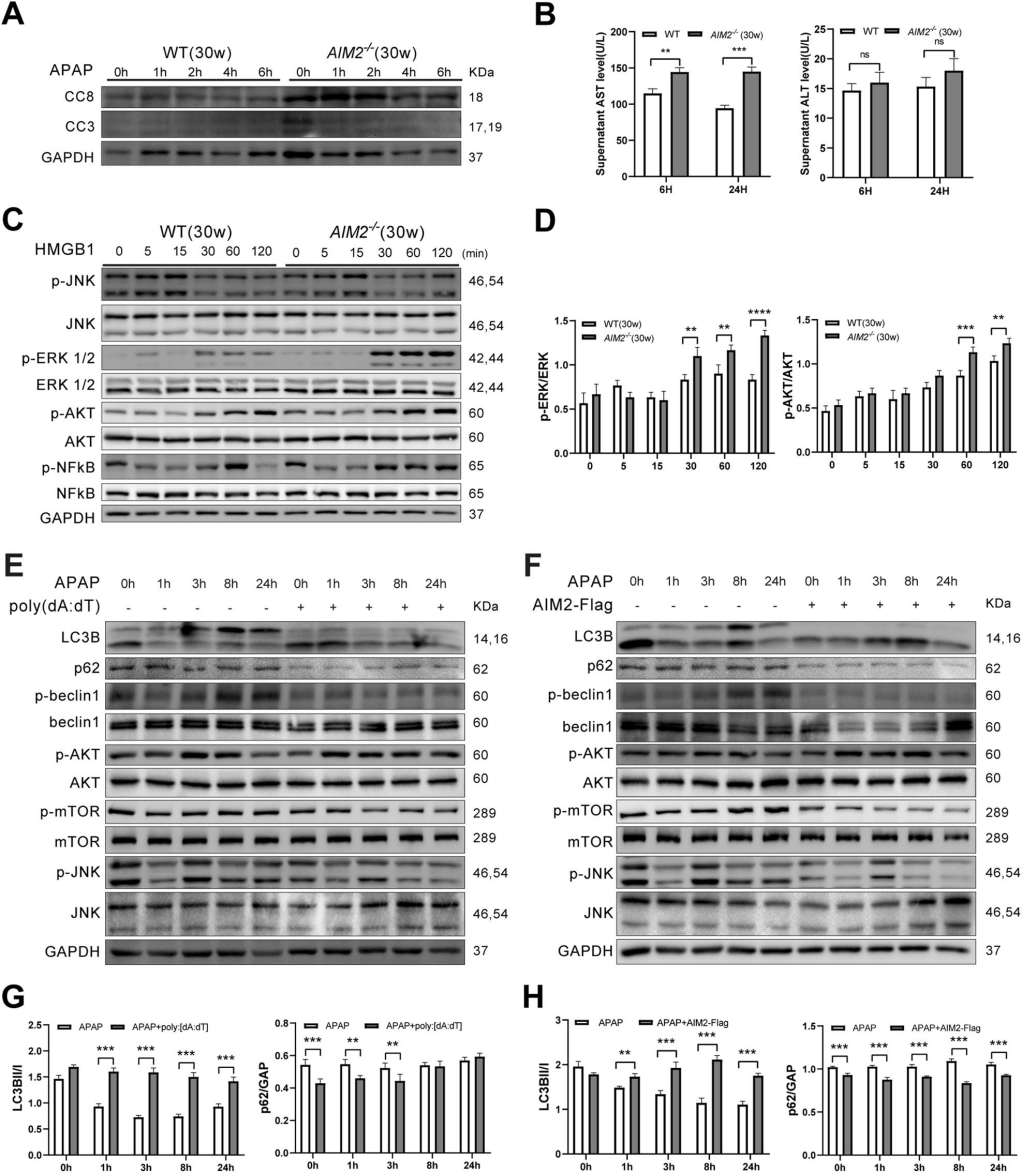
AIM2 deficiency promotes ERK signaling activation in macrophages and inhibits autophagy pathway activation in hepatocytes. **A** Western blot analysis of cleaved caspase-3 and 8 in primary hepatocytes from WT and *AIM2*^−/−^ mice aged 30 weeks stimulated with APAP (10 mM). **B** Primary hepatocytes from 30-week-old mice were treated with 10 mM APAP for 6 and 24 h, and culture supernatants were collected for AST and ALT measurement. **C**, **D** Bone marrow-derived macrophages (BMDMs) from 30-week-old mice were treated with HMGB1 (200 ng/mL), and cell proteins were collected for Western blotting. **E**–**H** TAMH cells were transfected with poly(dA:dT) or Flag-AIM2 for 24 h and then stimulated with 10 mM APAP. Cell lysates were obtained for Western blot analysis. Data represent the mean ± SEM of three independent experiments. **P < 0.01; ***P < 0.001; ****P < 0.0001.

The TUNEL assay revealed that *AIM2*^−/−^ mice exhibited more DNA fragments 24 h after APAP treatment (Fig. 2D). To investigate whether AIM2 deletion affected hepatocyte apoptosis, we examined the effects of APAP on primary hepatocytes from WT and *AIM2*^−/−^ mice in vitro. We observed a significant increase in cleaved caspase-8 (p18) expression levels in *AIM2*^−/−^ mice treated with APAP compared to WT mice, while cleaved caspase-3 expression was not significantly different (Fig. 6A). Furthermore, the AST level in the culture supernatant of primary hepatocytes from *AIM2*^−/−^ mice was significantly higher than in WT mice at 6 and 24 h after APAP stimulation (Fig. 6B), consistent with the results from previous animal experiments (Fig. 1E).

High mobility group protein 1 (HMGB1) is a common damage-associated molecular pattern (DAMP) that is often released as an important inflammatory mediator after cell damage [30]. Under mild oxidative conditions, extracellular HMGB1 acts as a pro-inflammatory cytokine, while intracellularly, it acts as an apoptosis-promoting agent [31]. We investigated the effects of HMGB1 on macrophage activation in WT and *AIM2*^−/−^ mice. We found that in response to HMGB1, bone marrow-derived macrophages (BMDMs) from *AIM2*^−/−^ mice showed higher activation of AKT and ERK signaling pathways compared to those from WT mice. However, we observed no alterations in p-NF-κB or p-JNK (Fig. 6C, D).

It has been reported that autophagy reduces oxidative stress and plays an anti-inflammatory role by eliminating damaged DNA fragments [32]. Moreover, AIM2 is degraded by p62-dependent selective autophagy [33]. Therefore, to investigate the role of AIM2 in the autophagy of hepatocytes after APAP-induced liver injury, we transfected TAMH cells with poly[dA:dT] or Flag-AIM2 overexpression plasmids, and then detected changes in proteins associated with autophagy signaling pathways after APAP stimulation in vitro, such as LC3B, p62, AKT, and mTOR. The results showed that when AIM2 was activated or overexpressed, the expression levels of p62, p-beclin1, and p-mTOR were significantly decreased, while p-AKT and LC3BII/I were markedly increased, indicating that the autophagy pathway was enhanced. Moreover, p-JNK was significantly decreased (Fig. 6E–H). Collectively, we suggest that AIM2 can promote autophagy and have a protective function in hepatic injury induced by APAP.

### Impact of AIM2 deficiency on monocyte and neutrophil infiltration in the liver

**Fig. 7:**
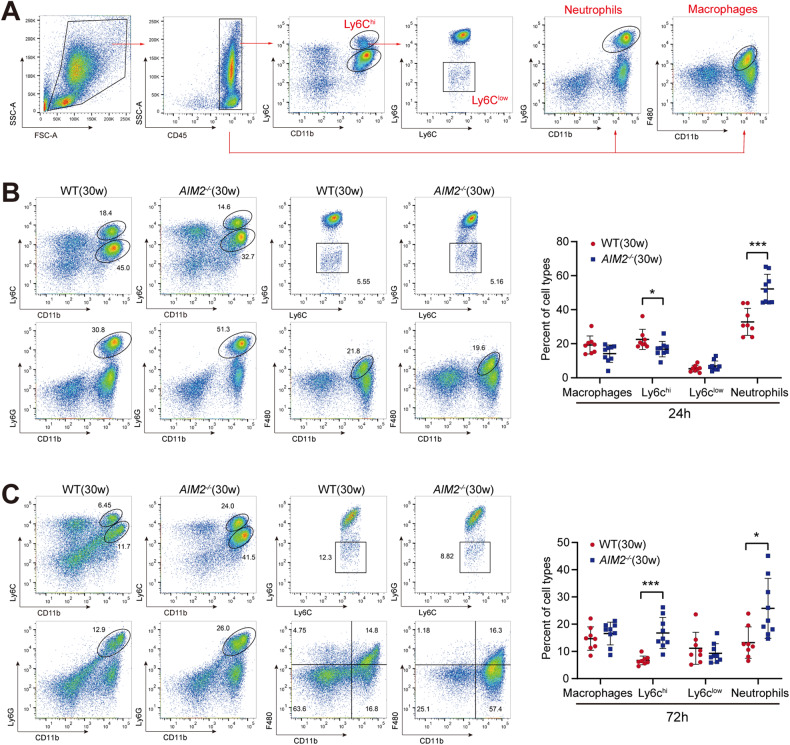
AIM2 deficiency influences the infiltration of inflammatory cells upon liver injury. **A** Fluorescence-activated cell sorting (FACS) strategy of immune cells. **B**, **C** Liver non-parenchymal cells (LNPCs) separated from WT and *AIM2*^−/−^ mice aged 30 weeks treated with APAP for 24 and 72 h for flow cytometry analysis. IMs were labeled as CD11b^+^ ly6c^high^ and CD11b^+^ ly6c^low^. Neutrophils and macrophages were labeled as CD11b^+^Ly6G^+^ and CD11b^+^F4/80^+^, respectively. Data represent the mean ± SEM of three independent experiments. *N* = 8–9 mice per group. *P < 0.05; ***P < 0.001.

Wide-scale hepatocyte necrosis leads to an aseptic inflammatory reaction and subsequent recruitment of inflammatory cells in the liver during APAP-induced hepatic injury [34]. Infiltrating monocytes (IMs) and neutrophils have been reported to mediate APAP-induced liver inflammation [35]. To investigate whether AIM2 affects the infiltration of immune cells after hepatic damage, we measured IMs and neutrophil infiltration in mouse liver after APAP administration using flow cytometry. Figure 7A shows the flow cytometry gating strategy for immune cells. Our analysis showed that compared to WT mice, neutrophil (CD11b^+^Ly6G^+^) recruitment in the liver of *AIM2*^−/−^ mice was significantly increased 24 h after APAP treatment and lasted until 72 h after treatment (Fig. 7B, C). Interestingly, in APAP-treated livers of *AIM2*^−/−^ mice, CD11b^+^ ly6c^high^ IMs (ly6c^hi^) were sparser at 24 h, but their proportion of recruitment was reversed at 72 h (Fig. 7B, C). Additionally, there was no significant difference in the infiltration of CD11b^+^ ly6c^low^ IMs (ly6c^low^) and macrophages (CD11b^+^F4/80^+^) (Fig. 7B, C).

### Rapamycin-induced autophagy activation ameliorates acute liver injury caused by APAP

**Fig. 8:**
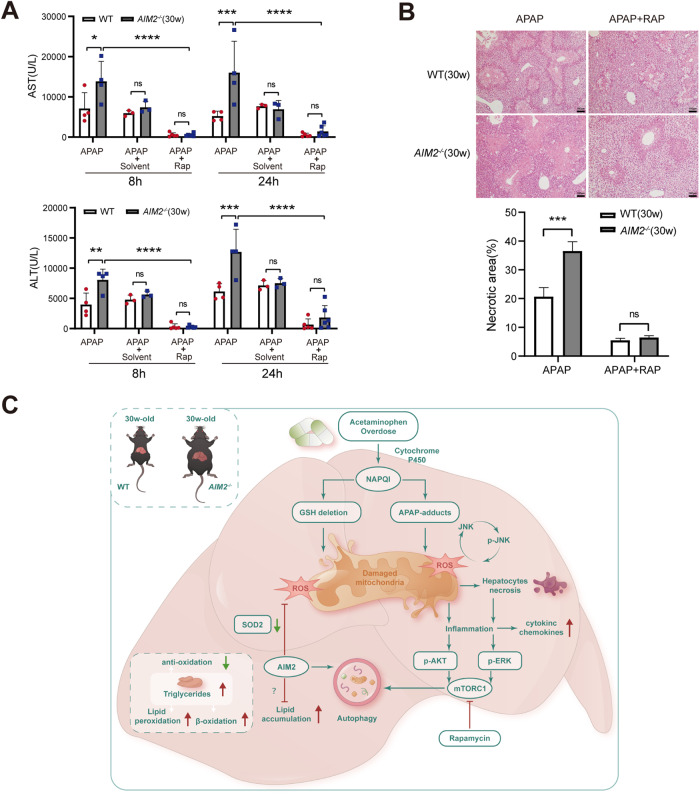
Activation of autophagy by Rap alleviates APAP-induced acute liver damage. **A** Mice aged 30–32 weeks were first treated with APAP for 2 h, followed by i.p. administration of RAP (2 mg/kg) or solvent (10% EtOH + 90% corn oil) to some mice, and ALT and AST serum levels were measured at 8 and 24 h. *n* = 4–6 mice per group. **B** Representative H&E staining images were shown, and necrotic parts quantification in the liver sections was performed. Scale bar, 100 µm. **C** Proposed molecular events of autophagy regulated by AIM2 in APAP-induced liver injury are summarized. Ns denotes no significance; *P < 0.05; **P < 0.01; ***P < 0.001; ****P < 0.0001.

In specific hepatic regions, excessive APAP can activate autophagy, which limits the expansion of necrosis into relatively normal hepatic regions [36]. Based on our APAP stimulation experiment of TAMH cells in vitro, we demonstrated that overexpression of AIM2 promotes autophagy and reduces the activation of APAP-induced JNK phosphorylation. We asked whether activation of autophagy could reduce hepatic injury caused by APAP in *AIM2*^−/−^ mice. To test this, we used rapamycin (Rap), a recognized autophagy agonist that inhibits the activation of mTOR. We studied its therapeutic effect in the APAP model and found that Rap protected against hepatotoxicity in both WT and *AIM2*^−/−^ mice, as confirmed by ALT levels (Fig. 8A) and H&E staining (Fig. 8B). Figure 8C illustrates the possible mechanisms by which AIM2 regulates autophagy in APAP-induced hepatic injury.

## Discussion

APAP overdose can cause acute hepatic failure and liver toxicity through various mechanisms, including the generation of nuclear DNA fragments, mitochondrial dysfunction, oxidative stress, and the formation of APAP metabolic protein adducts [37]. The cytoplasmic receptor AIM2 is known to recognize double-stranded DNA (dsDNA) from various sources, such as viruses, bacteria, and autologous cells [38]. Although AIM2 was first identified as a tumor suppressor in melanoma, most research on AIM2 has focused on its role in inflammasome activation and innate immune response against intracellular pathogens [39]. However, recent studies have revealed that AIM2 functions beyond inflammasome activation. For instance, two independent studies on colorectal cancer have shown that AIM2 regulates tumor development through inflammasome-independent pathways [8, 40]. Additionally, AIM2 interacts with DNA-dependent protein kinase (DNA-PK) to play a protective role against experimental autoimmune encephalomyelitis in microglia [41]. Here, we aimed to elucidate the role and signaling pathway of AIM2 in acute hepatic injury.

In the present study, no difference was observed in liver damage between WT and *AIM2*^−/−^ mice aged 6–8 weeks following excessive APAP treatment. This is consistent with previous report [42]. However, in mice aged 30–32 weeks, AIM2 deficiency was found to exacerbate acute liver injury, as evidenced by elevated serum ALT levels, H&E staining, and TUNEL staining. To investigate the pathway involved, we first evaluated APAP metabolism in mice. NAPQI, a highly active intermediate APAP metabolite produced mainly by the metabolism of cytochrome P450 enzymes CYP2E1 [43], is usually covalently bound with reduced GSH for detoxification [44]. We found no difference in the CYP2E1 protein levels between WT and *AIM2*^−/−^ mice, but *AIM2*^−/−^ mice exhibited lower levels of GSH than WT mice at 2 and 3 h after APAP administration, indicating that the detoxification ability of GSH as a covalently binding substrate of NAPQI was weakened after AIM2 knockout. Accordingly, NAPQI-protein adducts expression was significantly higher in *AIM2*^−/−^ mice than in WT mice, suggesting that AIM2 may play a protective role in APAP metabolism in the liver.

There have been reports indicating that a lack of AIM2 can lead to obesity and insulin resistance through the upregulation of the Ifi202b pathway [45]. This observation is consistent with the findings of our study, wherein the majority of *AIM2*^−/−^ mice were observed to be significantly heavier than the WT mice after a period of 30 weeks. Additionally, the weight and volume of the liver in *AIM2*^−/−^ mice were also significantly increased. The deficiency of AIM2 led to lipid accumulation, increased lipid peroxidation, and weakened ability to remove oxidative stress. The conjecture was confirmed by RNA-seq analysis. Lipid accumulation takes time, which could potentially explain the difference in hepatic damage observed in mice at 30 weeks.

The formation of APAP-protein adducts results in the recruitment of phosphorylated JNK into mitochondria, which further enhances mitochondrial damage and necrosis [22]. The pivotal role of JNK in liver damage caused by APAP has been confirmed by the protective effects of JNK inhibitors and knockout gene trials [46]. Therefore, we focused on detecting the expression of p-JNK in the livers of WT and *AIM2*^−/−^ mice after APAP overdose. Our study revealed that compared to WT mice, *AIM2*^−/−^ mice exhibited significantly elevated p-JNK expression. Notably, the overexpression of AIM2 in mouse hepatocytes, followed by APAP stimulation, led to decreased activation of p-JNK. Collectively, AIM2 may inhibit the activation of JNK in liver damage induced by APAP. Additionally, in the first 3 h following APAP treatment, ERK and P38 activation increased to varying degrees in *AIM2*^−/−^ mice. Therefore, during the metabolic period of APAP, AIM2 may inhibit the activation of the MAPK signaling pathway through some mechanism, leading to more severe liver injury after AIM2 knockout.

Lack of PDK4 has been shown to inhibit the creation of peroxynitrite and mitochondrial oxidative stress [24]. In DEGs analysis, *AIM2*^-/-^ mice showed higher PDK4 expression than WT mice. Therefore, we attempted to treat APAP hepatotoxicity with sodium dichloroacetate (DCA) (a PDK4 inhibitor). Mice were pretreated with DCA for 1 h and then injected with APAP. However, we found that more than half of the mice died at 6 h after APAP treatment, and there was no therapeutic effect (data not shown). Since AIM2 is a DNA receptor that senses aging DNA, we hypothesized that pretreatment to remove broken DNA in vivo could achieve a therapeutic effect and narrow the gap of liver injury between WT and *AIM2*^−/−^ mice. To test this hypothesis, we used NU7441, a highly effective and selective DNA-PK inhibitor, in a rescue trial. However, even with the NU7441 treatment, we observed no therapeutic effect (data not shown).

It has been reported that activating autophagy to remove APAP-protein adducts can protect against hepatic damage caused by APAP in mice [47, 48]. Our data also indicates differences in the expression of autophagy-related proteins, such as decreased expression of LC3B and enhanced activation of p-AKT, after AIM2 knockout. This finding was further confirmed by in vitro cell experiments, where overexpression of AIM2 enhanced the expression of LC3B and decreased the expression levels of p62, beclin1, and p-mTOR after APAP stimulation, indicating that AIM2 can directly regulate the autophagy pathway and play a promoting role. Excessive APAP leads to an increase in ROS and induces autophagy activation. Therefore, the weaker activation of autophagy in *AIM2*^−/−^ mice compared to WT mice may contribute to their increased susceptibility to APAP-induced liver injury. Finally, we treated mice with rapamycin, an autophagy inducer, and observed a significant decrease in APAP-induced hepatotoxicity in both *AIM2*^−/−^ and WT mice, confirming the protective role of autophagy in alleviating inflammatory damage.

In this study, we have provided evidence to demonstrate that the cytoplasmic sensor AIM2 plays a crucial role in the hepatic damage caused by APAP by mediating JNK activation, and also has a protective function in inflammation through autophagy. Unexpectedly, AIM2 played its role independently of the inflammasome. Further investigation is required to determine how AIM2 promotes lipid accumulation and lipid peroxidation with age. Overall, AIM2 can serve as a promising treatment target for acute liver failure with age.

## Materials and methods

### Mice

Male C57Bl6/J wild-type (WT) mice were obtained from the Nanjing Biomedical Research Institute of Nanjing University. AIM2 knockout (KO) mice on C57Bl6/J background were gifted by Professor Shuo Yang from Nanjing Medical University. The genotyping of *AIM2*^−/−^ mice was conducted using standard PCR, and the knockout efficiency of AIM2 was shown in supplementary Fig. 1. The following primers were utilized in the process: CCAGTGTTTCTCAACTGTACTGCTAT, TAGGAGTGCCCTCCCTTAATG, TTGGAGACAGACTCTGGTGAAG. The expected sizes of the amplicons are as follows: wild type (WT): 197 bp, *AIM2*^−/−^: 397 bp. The mice were aged 6–8 weeks and 30–32 weeks. Researchers did not conduct blinding for the groups of mice. The sample size was not determined using statistical methods. Adequate sample size was determined based on the replicability of the experiments. In the study, each mouse was individually assigned a unique numerical identifier, and subsequent grouping of animals was performed using a random number table. The standard 12-h light/dark cycle was used for the mice. All animal procedures were approved by the Laboratory Animal Core Facility of Nanjing Medical University (approval No. IACUC-1912021).

### Induction and assessment of acute hepatic injury

Acute hepatic injury was induced in mice by administering intraperitoneal injections of APAP (acetaminophen) or phosphate solution (PBS) after fasting the mice for 16–18 h. The dosage of APAP was 300 mg/kg and it was dissolved in warm saline. After receiving the injections, the mice were either sacrificed at different time intervals to collect liver specimens for histology or frozen in liquid nitrogen and kept at −80 °C for later use. Blood was also collected through retro-orbital puncture and serum levels of aspartate aminotransferase (AST) and alanine aminotransferase (ALT) were measured using an automatic chemical analyzer. In a rescue experiment, some mice were supplemented with rapamycin (2 mg/kg, i.p., HY-10219, MedChemExpress, China) at 2 h after APAP injection. Rapamycin was dissolved in a mixture of 10% ethanol and 90% corn oil. The control group received a solvent injection instead. Serum and liver tissue were collected at a specified time after injection.

### Quantitative real-time PCR

TRIzol reagent was used to extract total RNA from liver tissues, which was then subjected to cDNA synthesis using RT SuperMix (Vazyme #R323). The ChamQ Universal SYBR qPCR Master Mix (Vazyme #Q711) and the Step One Plus Real-Time PCR System (Thermo Fisher Scientific, Waltham, MA, USA) were used to perform RT-qPCR to detect the relative mRNA levels of the target genes. The mRNA levels were normalized to the expression level of Gapdh using the 2^−ΔΔCt^ method. The primers used for the qPCR are listed in supplementary Table 1.

### Histological analysis

The liver tissue specimens that had been embedded in paraffin were sectioned into 4 μm segments and stained with hematoxylin and eosin (H&E) to evaluate cell death patterns. To detect protein adducts, a sheep anti-APAP polyclonal antibody (#0016-0104, Bio-Rad, Düsseldorf, Germany) was utilized. The stained tissue samples were examined under a light microscope (Olympus IX51, Japan). The pathological tissue section images were analyzed using Image J (1.51j8).

### Western blotting

Proteins were extracted from mouse livers or cultured cell specimens using a previously described method [28]. A 25 μg sample of total protein was separated on SDS-PAGE and then transferred onto a PVDF membrane. The membrane was blocked with 5% bovine serum albumin (BSA) at room temperature for 2 h. Primary antibodies including anti-AIM2 (#13095 s), anti-total AKT (#4691), anti-GAPDH (#5174), anti-total ERK (#4695), anti-p-JNK (#9251), anti-total JNK (#9258), anti-p-p38 (#9211), anti-total p38 (#9212), anti-p-AKT(Ser473) (#9271), anti-p-AKT(Thr308) (#13038), anti-p-STAT3 (#9131), anti-total STAT3 (#9139), anti-p-NF-κB (#3033), cleaved caspase-8 (#8592), anti-total NF-κB (#8242), cleaved caspase-3 (#9664), anti-p-ERK (#4370), and PCNA (#2586) were obtained from Cell Signaling Technology (Beverly, MA, USA). Antibodies against Cytochrome b (CYTB) (#55090-1-AP), Mitochondrial transcription factor A (TFAM) (#22586-1-AP), VDAC (#55259-1-AP), tubulin (#11224-1-AP), and NADH dehydrogenase subunit 1 (ND1) (#19,703-1-AP), were obtained from Proteintech Group, Inc (Wuhan, China). Anti-CYP2E1 antibody (BML-CR3271-0100) was obtained from Enzo Life Sciences (NY, USA). The antibodies were diluted according to the manufacturer’s instructions. The membrane was incubated with the primary antibody overnight at 4 °C, followed by detection with horseradish peroxidase-conjugated secondary antibody.

### Isolation of mitochondrial proteins

Mitochondria were isolated from 293 T cells using a mitochondria isolation kit (Cat# C3601; Beyotime Biotechnology). Briefly, cells was collected, washed, and homogenized in mitochondrial separation reagent according to the manufacturer’s protocol. The mitochondrial and cytoplasmic proteins were quantified and subjected to further analysis.

### Mitochondrial stability analysis

To measure the relative mitochondrial DNA/nuclear DNA (mtDNA/nDNA) ratio, total DNA was extracted from mouse livers using the DNA Mini Preparation Kit (Cat# D0063; Beyotime Biotech, China). The primers for mtDNA and nDNA are listed in supplementary Table 1.

### Glutathione (GSH) measurement

Utilizing a colorimetric Glutathione Assay Kit (CS0260, Sigma-Aldrich), total hepatic GSH was measured following the manufacturer’s instructions. First, the frozen hepatic tissue was deproteinized using a 5% solution of 5-sulfosalicylic acid. Then, dithio-nitrobenzoic acid (DTNB) was utilized to measure total glutathione, and spectrophotometric measurements were taken at 412 nm.

### Immunofluorescence

293 T cells were seeded onto a 24-well plate that was pre-coated with coverslips. The overexpression plasmid, Flag-AIM2, was transfected into the 293 T cells using Lipofectamine 3000 (Invitrogen). After 24 h, the cells were incubated with 50 nM of Mito-tracker Red CMXRos (Cat# C1035; Beyotime, China) for 30 min. Following fixation, the cells were blocked overnight at 4 °C with an Anti-Flag antibody (Cat# GB15938; Servicebio, China). The following day, the cells were incubated with Alexa Fluor 488-conjugated fluorescent secondary antibodies (Cat# A0428; Beyotime, China), and the nuclei were stained with DAPI. Images were captured using an Olympus IX51 microscope.

### The enzyme-linked immunosorbent assay (ELISA)

Following the manufacturer’s instructions, serum was collected from the mice and IL-6 levels were measured using an ELISA kit from BioLegend (San Diego, CA, USA) by following the manual.

### Terminal deoxynucleotidyl transferase dUTP nick end-labeling (TUNEL) assay

The One Step TUNEL Apoptosis Assay Kit (Beyotime, Beijing, China) was used to perform TUNEL staining for cell death according to the manufacturer’s protocol.

### Hepatic sinusoidal endothelial cell permeability

One day after APAP administration, mice were injected intraperitoneally with Evans blue dye (20 mg/kg, Sigma-Aldrich, USA). After 4 h, the liver was perfused with HBSS in situ, removed, and placed in formamide (4 mL/g liver), followed by overnight incubation at 45 °C to extract the dye. The amount of Evans blue dye in the extract solution was determined by measuring the absorbance at 630 nm, and the permeability was calculated and expressed as μg/g liver.

### Cell culture and transfection

The transforming growth factor-transgenic mouse hepatocytes (TAMH cells) were grown in DMEM/F12 with 10% fetal bovine serum (FBS; Gibco, NY, USA) and 1% penicillin/streptomycin (Thermo Scientific, Waltham, MA, USA), and were preserved with 5% CO_2_ in a humid incubator at 37 °C. The cells were a kind gift from Dr. Cynthia Ju of UTHealth in Houston, Texas. AIM2 cDNA plasmid (MG58612-NF) was obtained from Sino Biological (Beijing, China). Poly [dA:dT] was acquired from InvivoGen (CA, USA). For transfection, Lipofectamine 3000 (Invitrogen) was utilized according to the instructions.

### Isolation and culture of bone marrow-derived macrophages (BMDM)

The tibia and femur of the mice were collected after they were euthanized using carbon dioxide. RPMI 1640 medium was used to flush the bone marrow, and the cell suspension was filtered through a 70-µm cell filter (BD Falcon, Bedford, MA, USA). The red blood cells were removed using a red blood cell lysis solution after centrifugation at 500 × *g*. The cells were then cultured in RPMI 1640 medium containing 10 ng/mL of macrophage colony-stimulating factor (M-CSF) and 10% FBS for three days. Afterward, all dishes were supplemented with 4 mL of RPMI 1640 medium containing 14 ng/mL of M-CSF and 10% FBS and cultured for an additional three days. The cells were then seeded (5 × 10^6^/ well) onto cell culture plates for various treatments.

### Liver non-parenchymal cells (NPCs) isolation

Liver non-parenchymal cells (LNPCs) were isolated according to a pre-established protocol [49]. Briefly, the liver was dissected and homogenized using Hank’s balanced salt solution (HBSS) with 0.5% FBS after perfusion with HBSS with EGTA. The resulting single-cell suspensions were filtered through a 100-µm cell strainer (BD Falcon, Bedford, MA, USA). Liver NPCs were then isolated using 35% Percoll (Sigma-Aldrich, St. Louis, MO, USA). Erythrocyte lysis buffer was used for further lysis of erythrocytes. Finally, cells were resuspended in HBSS with 2% FBS for fluorochrome-conjugated staining.

### Primary hepatocytes isolation

As previously described, primary hepatocytes were isolated using the following protocol [50]. Briefly, mice were euthanized using carbon dioxide and the livers were perfused with Ca^2+^ and Mg^2+^-containing HBSS buffer in situ for 5 min. This was followed by perfusion for 2 min with a Ca^2+^, Mg^2+^-free HBSS buffer, and then for 10 min with a 0.04% solution of type IV collagenase (Sigma-Aldrich). After digestion, the liver was gently agitated in a 50 mL Falcon tube containing Williams E medium to disperse the cells. The liver cells were then separated from the resulting single-cell suspension by filtration through a 100-μm cell strainer and centrifuged at 40×*g* for 3 min. Cell viability was assessed using trypan blue staining. For subsequent experiments, the liver cells were seeded onto six-well petri plates containing William’s E medium supplemented with 10% FBS.

### Flow cytometry

To prevent non-specific binding, freshly isolated liver NPCs were treated with mouse Fc receptor blockers. Fluorochrome-conjugated antibodies were then added to the cells and incubated. These antibodies included APC-conjugated anti-mouse F4/80 (clone BM8, #17-4801-82, eBioscience, San Diego, CA, USA), FITC-conjugated anti-mouse Ly6C (clone AL21, #553104, BD Biosciences, San Jose, CA, USA), PE-vio770 conjugate anti-mouse CD11b (clone M1/70, #25-0112-82, eBioscience), APC Cyanine7 conjugated anti-mouse CD45 (clone 30F11, # 103116, Biolegend, San Diego, CA, USA), and PE-conjugated anti-mouse Ly6G (clone 1A8, #127608, Biolegend). Data analysis was performed using the BD FACSCanto II flow cytometer and the FlowJo (V10) software.

### RNA-sequencing and data analysis

Male WT and *AIM2*^−/−^ mice at 8 and 30 weeks of age, with three mice in each group, were used in this study. One day after APAP treatment, hepatic specimens were obtained and immediately flash-frozen in liquid nitrogen in 1.5 mL RNase-free EP tubes. The samples were transported on dry ice for total RNA isolation and RNA sequencing analysis. The cDNA library was constructed and sequenced using the BGISEQ-500 platform by the Beijing Genomics Institute (BGI). The raw RNA-seq data has been deposited in the Sequence Read Archive (SRA) (https://www.ncbi.nlm.nih.gov/sra) under the accession code PRJNA1065821. BGI developed a platform for implementing bioinformatics processes, including data screening, marker transcript prediction, differential gene expression analysis, Gene Ontology (GO), Kyoto Encyclopedia Genes and Genomes (KEGG) pathway analysis, and Gene Set Enrichment Analysis (GSEA).

### Statistics

The mean ± SEM was used to represent all values, and the GraphPad Prism program (version 8.0.2) was used for analysis. A two-tailed unpaired Student *t*-test or ANOVA was used to evaluate the statistical significance between groups, depending on the number of groups being compared. The variance is relatively consistent or stable among the groups being compared. All experiments were performed at least three times. *P* values were used to indicate the level of statistical significance, with *P* < 0.05 indicating significance, ** indicating *P* < 0.01, and *** indicating *P* < 0.001.

### Supplementary information

Supplementary Figures and tables

Original Data File

## Data Availability

The additional data and materials during the current study are available from the corresponding author on reasonable request.
