# Supplementary material for: AIM2 regulates autophagy to mitigate oxidative stress in aged mice with acute liver injury
Source: Cell Death Discov. 2024 Mar 1;10:107. doi: 10.1038/s41420-024-01870-2 (PMC10907373; doi:10.1038/s41420-024-01870-2)
Supplement: Supplementary file 1 — Supplementary Figures and tables [file 41420_2024_1870_MOESM1_ESM.pdf]

Supplementary Figure 1

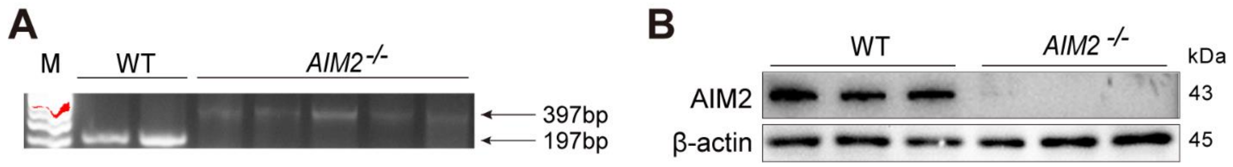

Fig. S1. The knockout efficiency of AIM2 gene. (A) PCR genotyping of *AIM2*$^{-/-}$mice. The brand shows PCR products characteristic for WT (197bp) and *AIM2*$^{-/-}$ (397bp) mice. M: marker. (B) AIM2 protein was determined in the livers from WT and *AIM2*$^{-/-}$ mice by western blotting.

Supplementary Figure 2

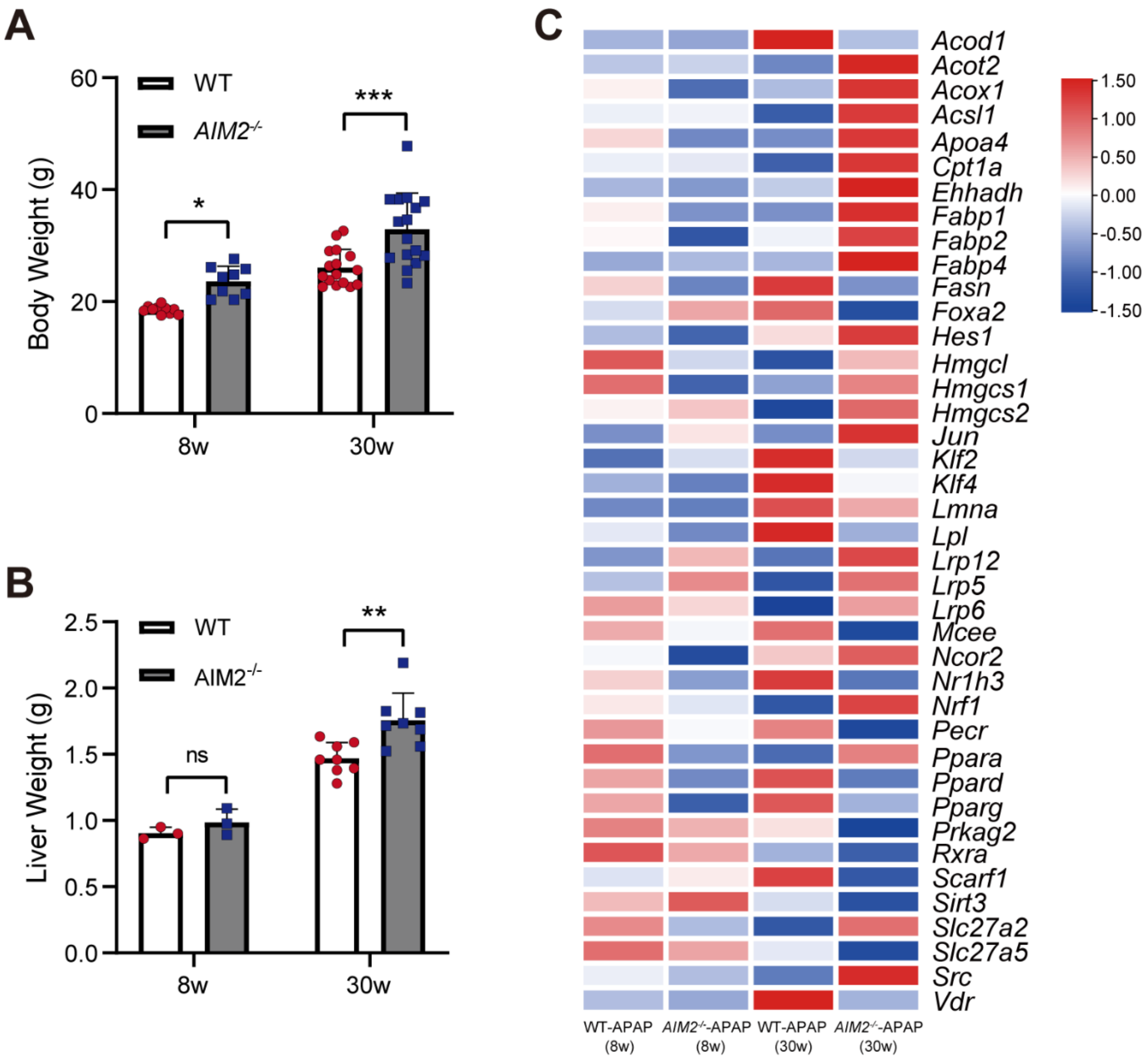

Fig. S2. AIM2 correlates with lipid metabolism. (A) Body weight and (B) liver weight of WT and *AIM2*$^{-/-}$ mice aged 6–8 and 30–32 weeks. Data represent the mean ± SEM. N = 8–10 mice per group. (C) Heat map displaying the differentially expressed genes (DEGs) related to lipid metabolism in the four groups. Each row represents the relative mRNA expression (red, high; blue, low).

Supplementary Table 1 Primers used for qPCR

	Forward Sequence (5'-3')	Reverse Sequence (5'-3')
GAPDH	CATCACTGCCACCCAGAAGACTG	TGCCAGTGAGCTTCCCGTTCAG
IL-1α	ACGGCTGAGTTTCAGTGAGACC	CACTCTGGTAGGTGTAAGGTGC
IL-1β	TGGACCTTCCAGGATGAGGACA	GTTCATCTCGGAGCCTGTAGTG
IL-18	GACAGCCTGTGTTCGAGGATATG	TGTTCTTACAGGAGAGGGTAGAC
TNF-α	GGTGCCTATGTCTCAGCCTCTT	GCCATAGAACTGATGAGAGGGAG
IL-6	TACCACTTCACAAGTCGGAGGC	CTGCAAGTGCATCATCGTTGTTC
MCP1	TGTACCATGACACTCTGCAAC	CAACGATGAATTGGCGTGGAA
AIM2	AGGCTGCTACAGAAGTCTGTCC	TCAGCACCGTGACAACAAGTGG
mitochondrial DNA	GTGGAATACGCCAGTGAGAAGC	CAACTTGCTGGCACAGATGAGC
nuclear DNA	TGCTGTCTCCATGTTTGATGTATCT	TCTCTGCTCCCCACCTCTAAGT
