# Supplementary figures and images for: AIM2 regulates autophagy to mitigate oxidative stress in aged mice with acute liver injury
Source: Cell Death Discov. 2024 Mar 1;10:107. doi: 10.1038/s41420-024-01870-2 (PMC10907373; doi:10.1038/s41420-024-01870-2)

Fig.1D

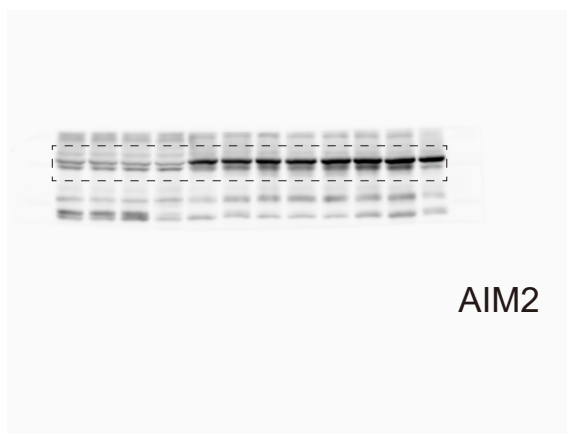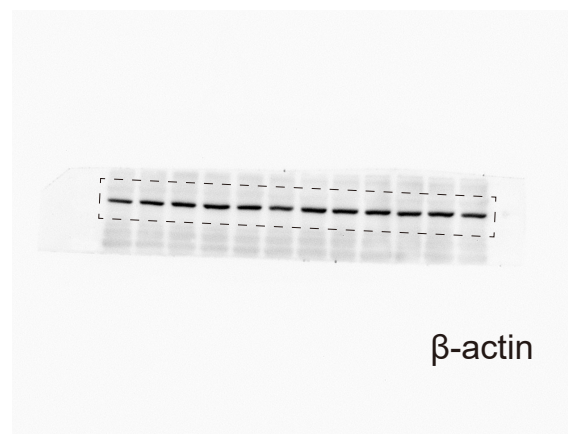

Fig.2A

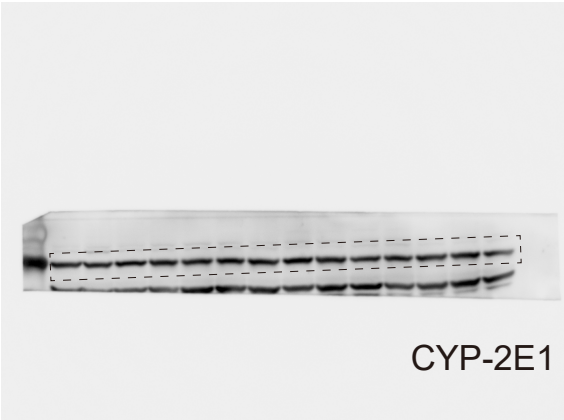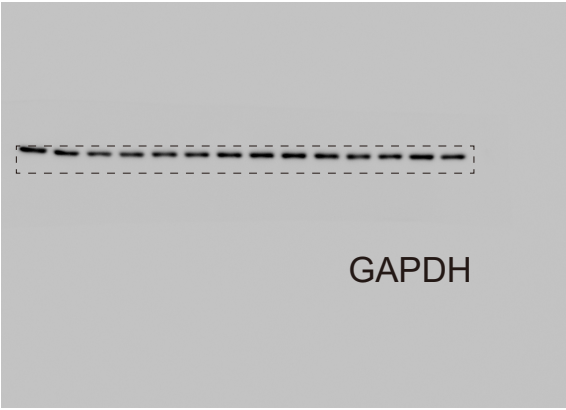

Fig.2E

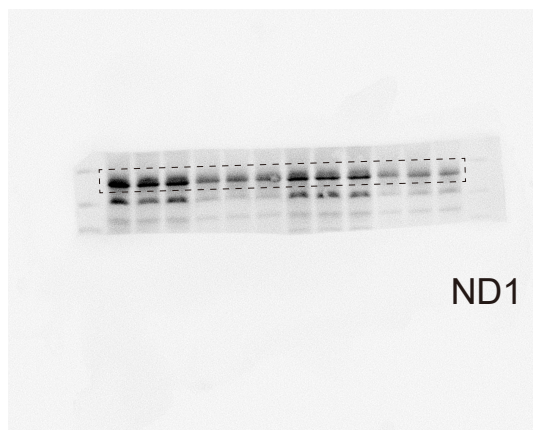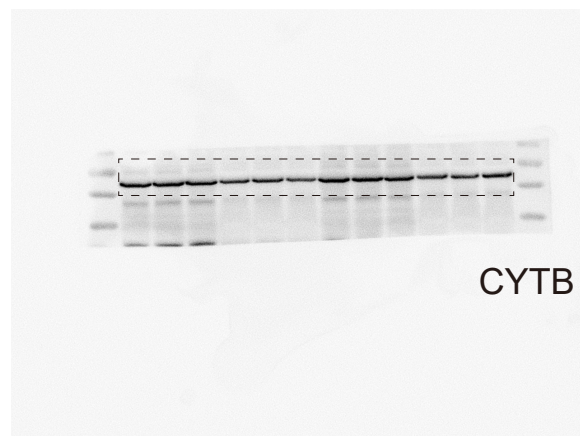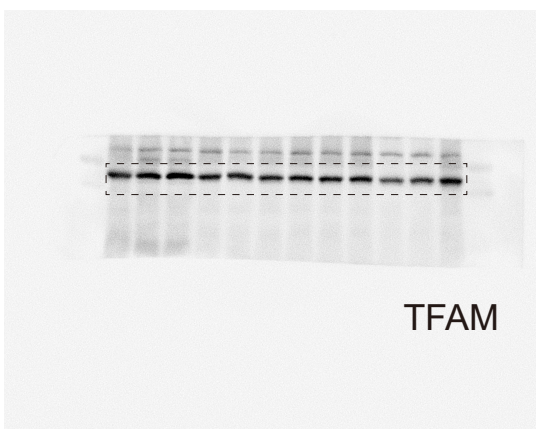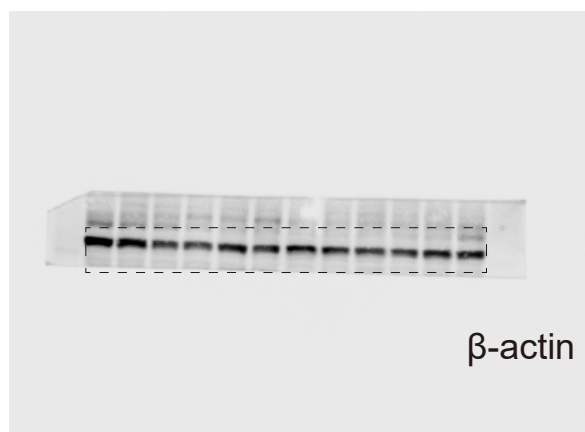

Fig.2F

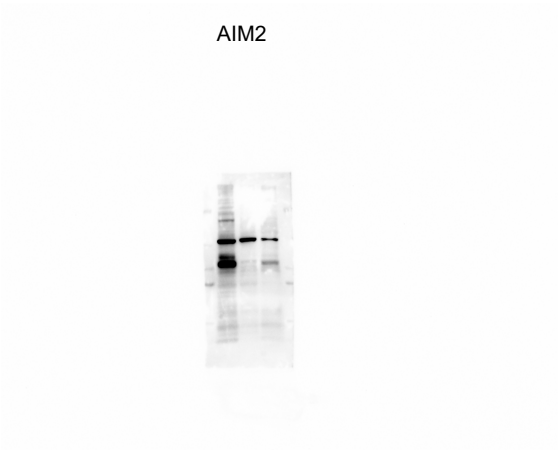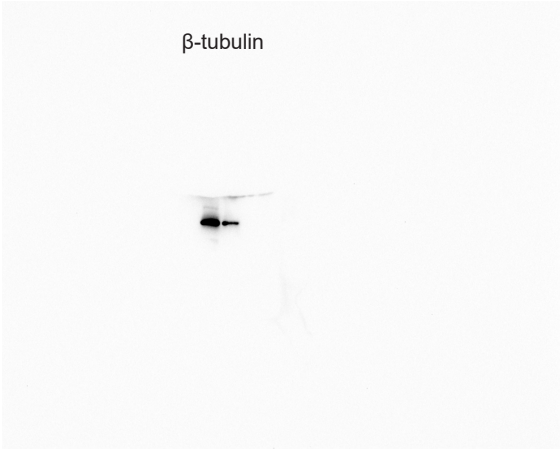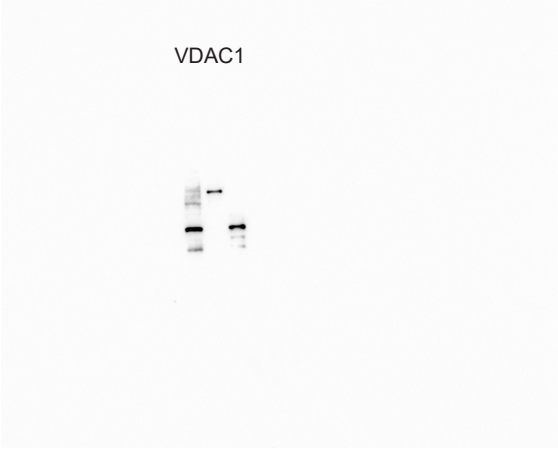

Fig.5A

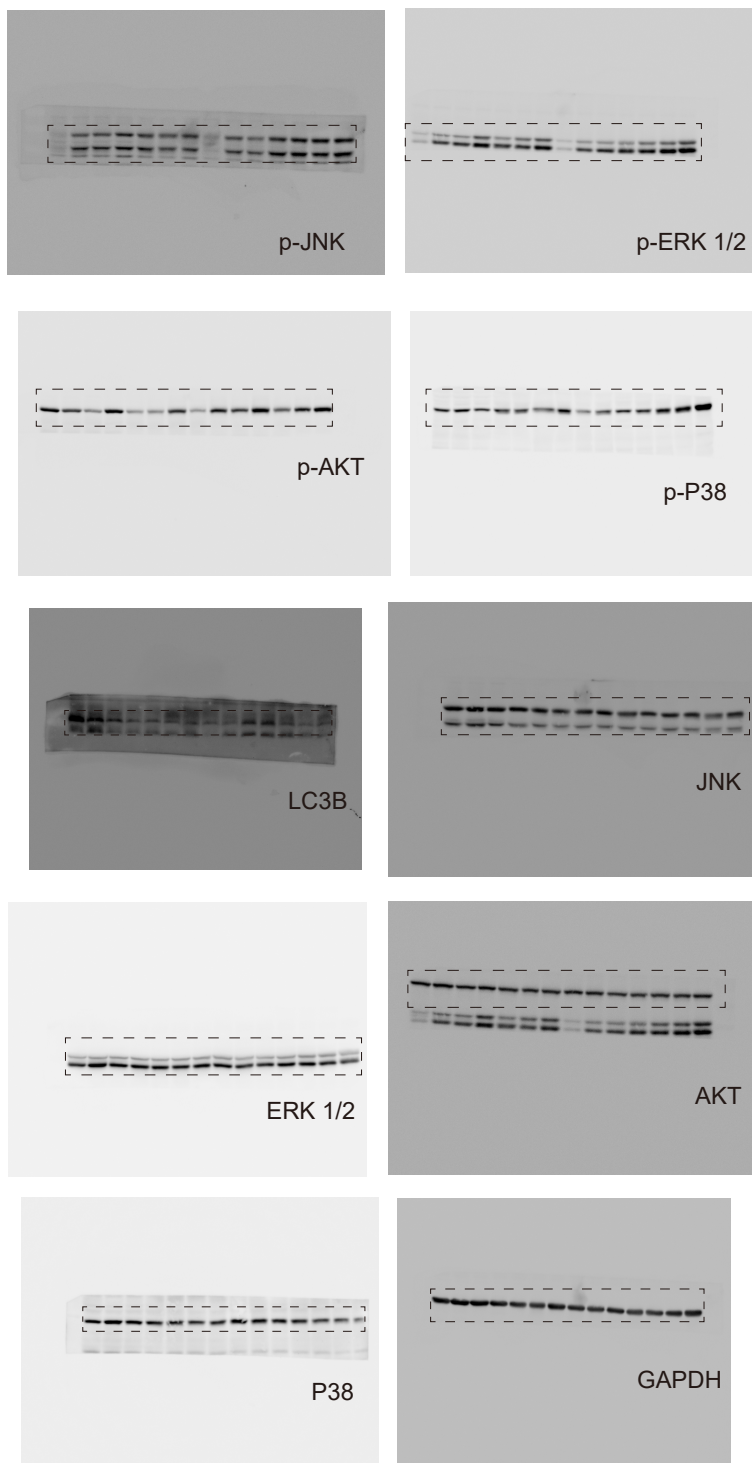

Fig.5C

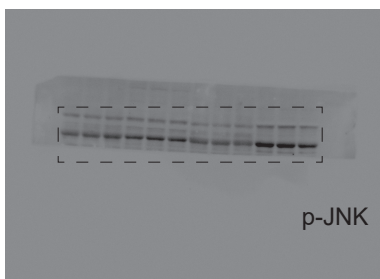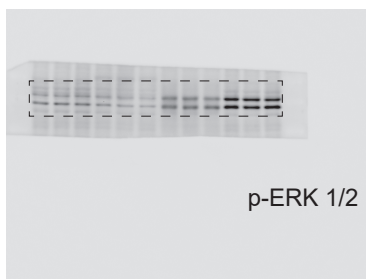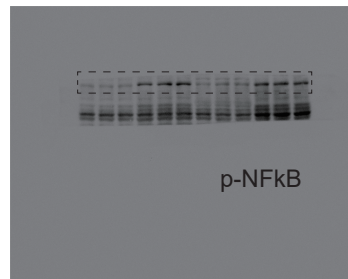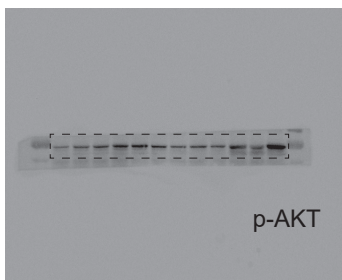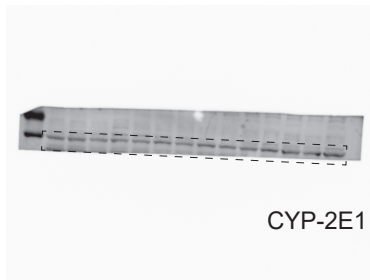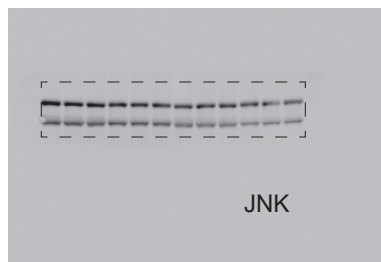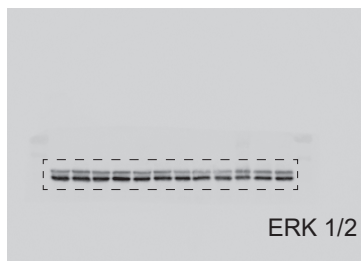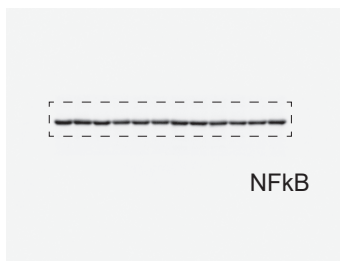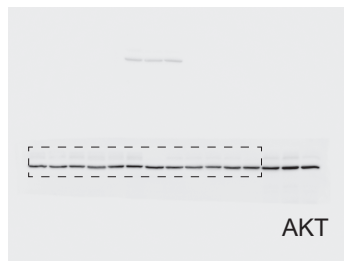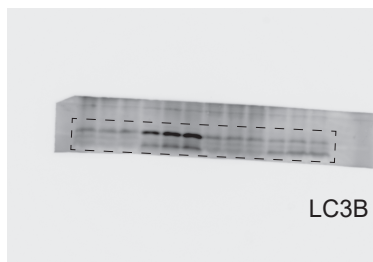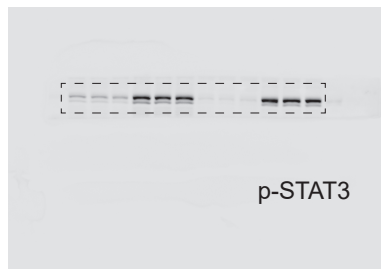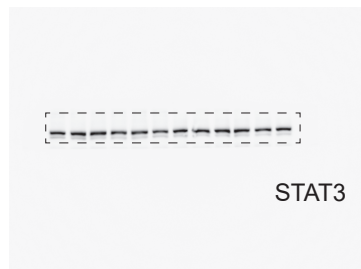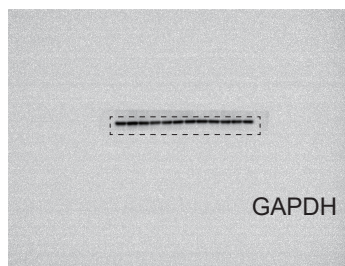

Fig.5D

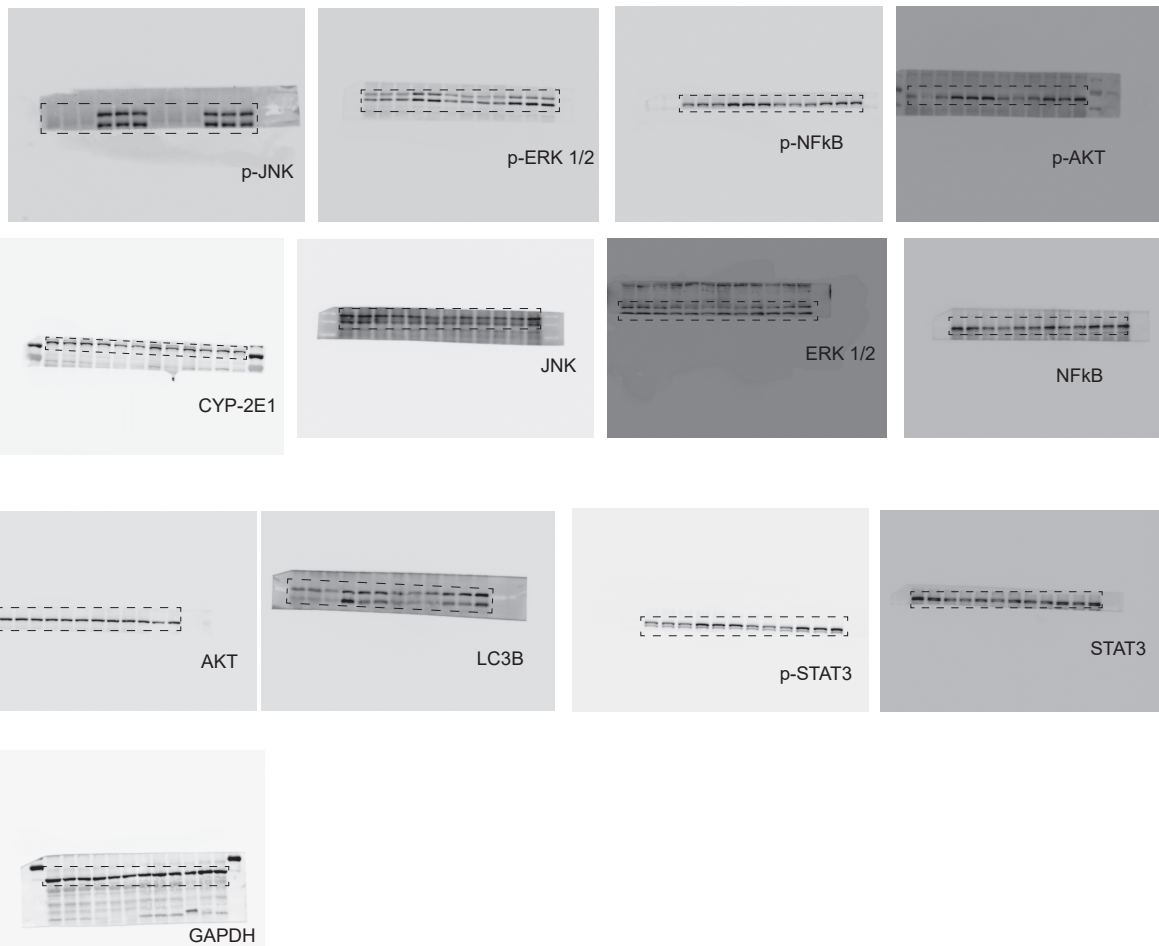

Fig.5G

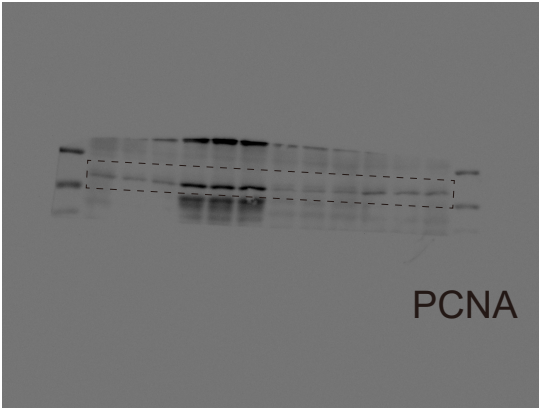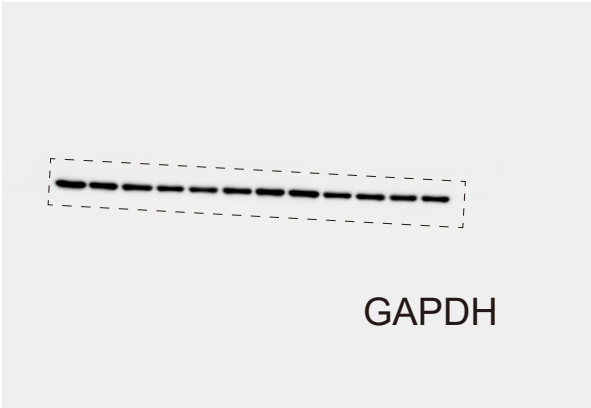

Fig.6A

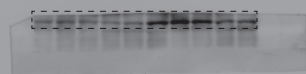

CC8

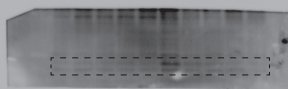

CC3

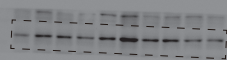

GAPDH

Fig.6C

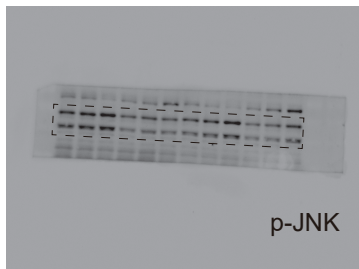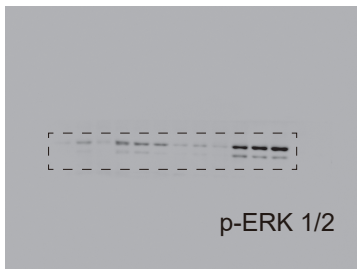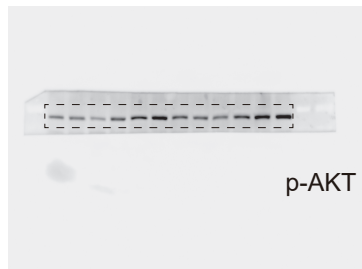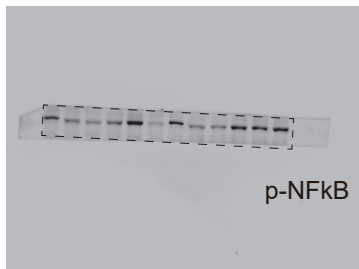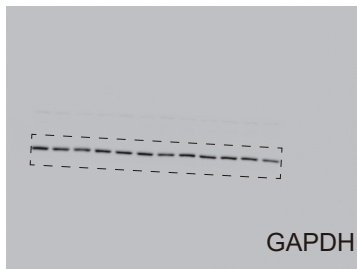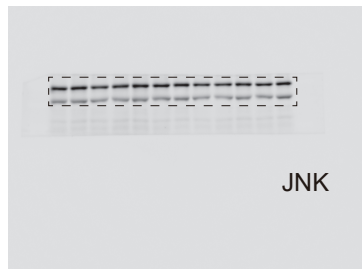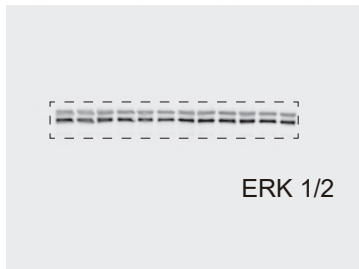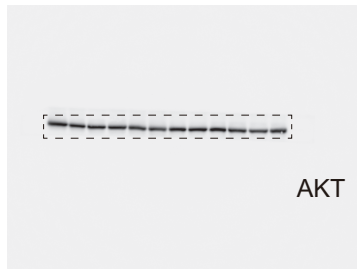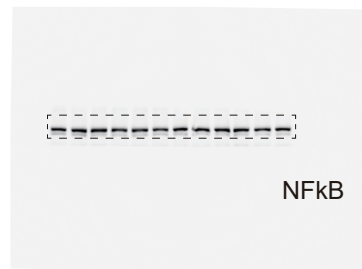

Fig.6E

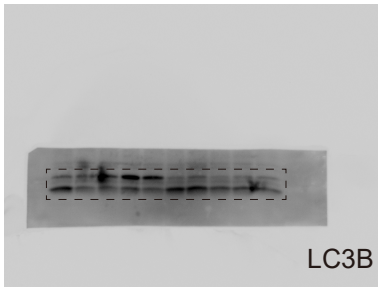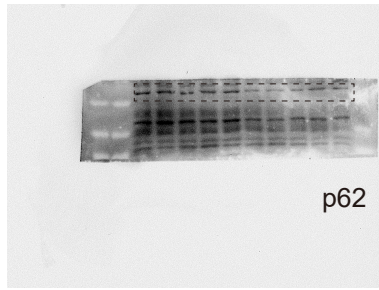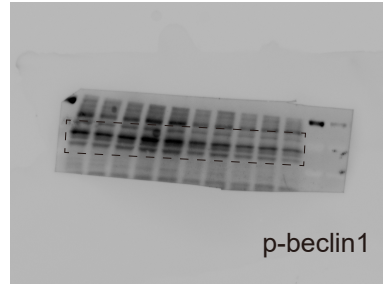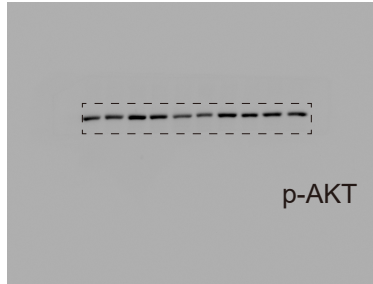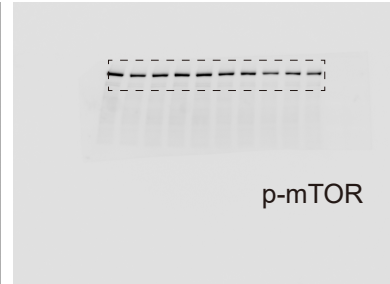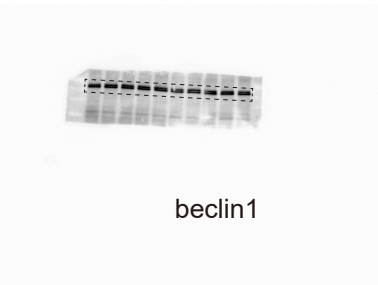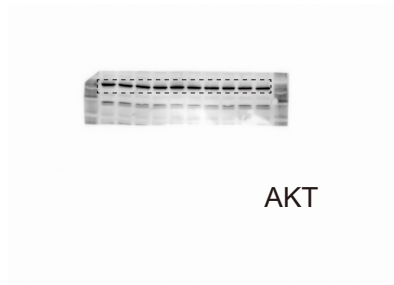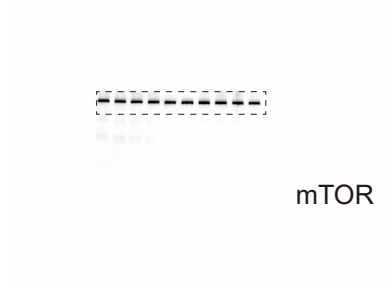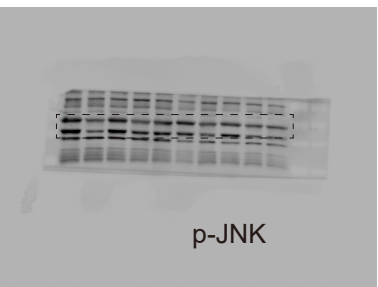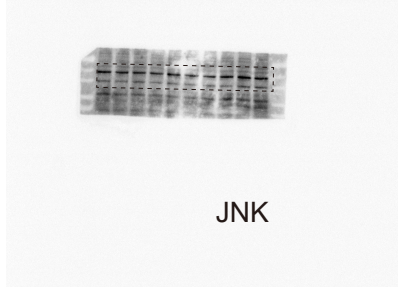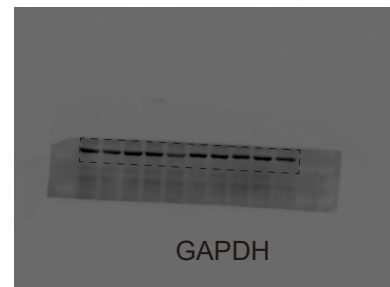

Fig.6F

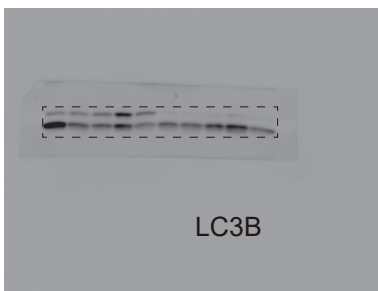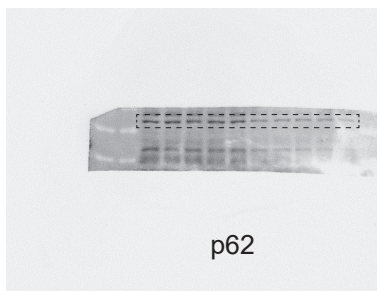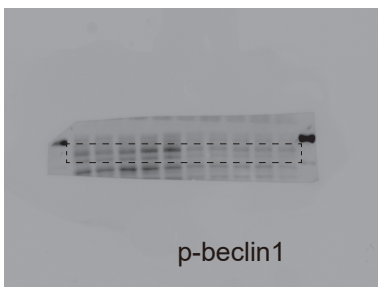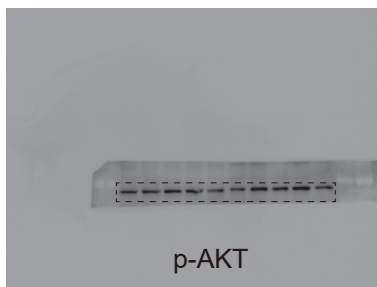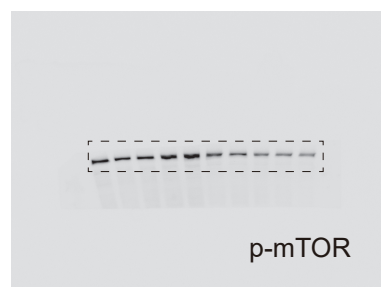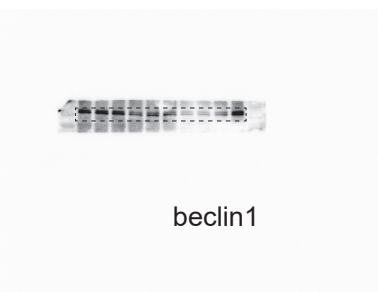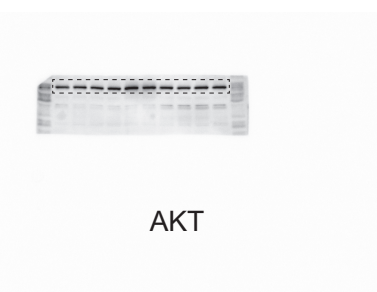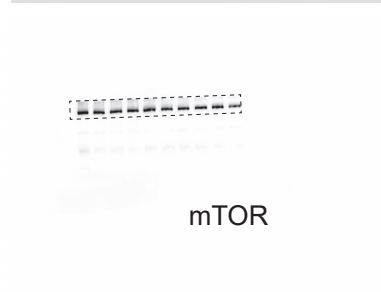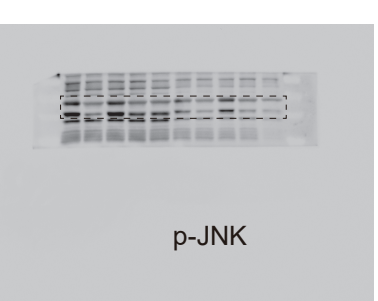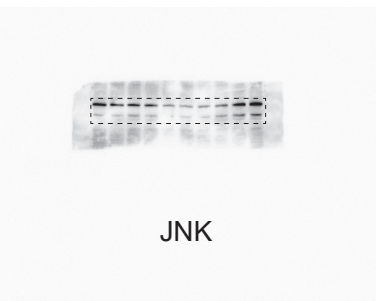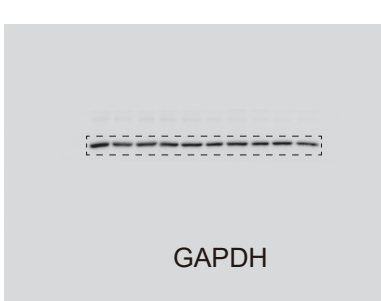

supplementary Figure 1

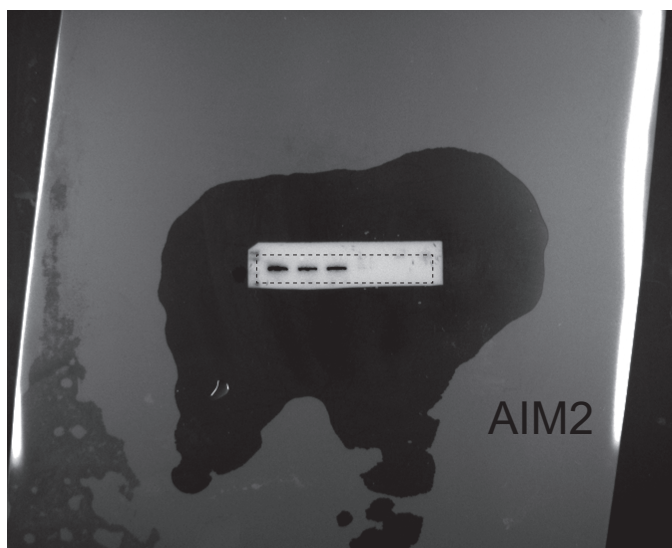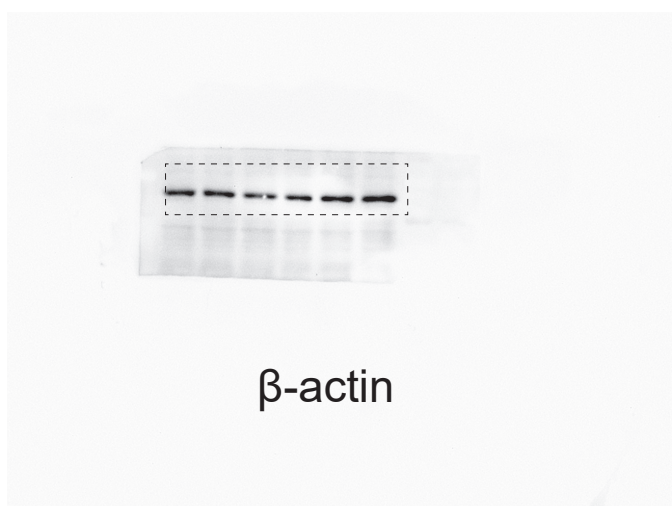

Supplement: Supplementary file 2 — Original Data File [file 41420_2024_1870_MOESM2_ESM.pdf]
